# Systematic Assessment of Fragment Identification for Multitarget Drug Design

**DOI:** 10.1002/cmdc.202000858

**Published:** 2021-02-04

**Authors:** Steffen Brunst, Jan S. Kramer, Whitney Kilu, Jan Heering, Julius Pollinger, Kerstin Hiesinger, Sven George, Dieter Steinhilber, Daniel Merk, Ewgenij Proschak

**Affiliations:** ^1^ Institute of Pharmaceutical Chemistry Goethe University Max-von-Laue Str. 9 60438 Frankfurt Germany; ^2^ Fraunhofer Institute for Translational Medicine and Pharmacology ITMP Theodor-Stern-Kai 7 60596 Frankfurt Germany

**Keywords:** differential scanning fluorimetry, fragment-based drug design, multitarget drugs, polypharmacology

## Abstract

Designed multitarget ligands are a popular approach to generating efficient and safe drugs, and fragment‐based strategies have been postulated as a versatile avenue to discover multitarget ligand leads. To systematically probe the potential of fragment‐based multiple ligand discovery, we have employed a large fragment library for comprehensive screening on five targets chosen from proteins for which multitarget ligands have been successfully developed previously (soluble epoxide hydrolase, leukotriene A4 hydrolase, 5‐lipoxygenase, retinoid X receptor, farnesoid X receptor). Differential scanning fluorimetry served as primary screening method before fragments hitting at least two targets were validated in orthogonal assays. Thereby, we obtained valuable fragment leads with dual‐target engagement for six out of ten target combinations. Our results demonstrate the applicability of fragment‐based approaches to identify starting points for polypharmacological compound development with certain limitations.

## Graphical abstract

**Thinking outside the pharmacophore**: This study systematically investigates the potential of fragment‐based multiple ligand discovery. We employed a large fragment library for comprehensive screening on five targets and obtained valuable fragment leads with dual‐target engagement for six out of ten target combinations.

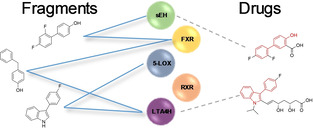

Designed polypharmacology has markedly gained importance in the past decade with increasing numbers of scientific publications and FDA approvals of designed multitarget drugs (DMLs).^[1]^ DMLs offer certain advantages over the “traditional” selective ligands: improved efficacy from synergistic target engagement, as well as better safety and patient compliance compared to polypharmaceutical treatment.[^2^, ^3^] However, the design of DMLs with desirable properties can be a challenging task.^[4]^ DMLs often comprise poor ADME properties resulting from high molecular weights as a consequence pharmacophore linkage as the simplest strategy to obtain DMLs by joining of pharmacophores via a molecular linker. Thus, pharmacophore fusion for two (or more) targets in a common molecular framework or identification of a merged pharmacophore is a more attractive approach to design DMLs with favorable profile. Morphy and Rankovic proposed the concept of fragment‐based design of DMLs with merged pharmacophore.^[5]^ According to this strategy, a low‐molecular‐weight fragment binding to the desired multiple targets is optimized for potency simultaneously on the target proteins. Several studies have demonstrated the feasibility of this approach.[^6^, ^7^, ^8^] However, the key step of this concept is the identification of a suitable molecular fragment to serve as a starting point. Studies by Hann et al.^[9]^ and Hopkins et al.^[10]^ imply that there is a high probability to identify such fragments from screening due to the fact that binding promiscuity increases with lower molecular weight.

In this study, we aimed to systematically probe the feasibility of identifying fragment hits for multitarget drug discovery. For this, we chose five proteins that have been successfully targeted by DMLs previously to ensure that multitarget ligands for these proteins are possible. Our target choice covered the enzymes 5‐lipoxygenase (5‐LOX), soluble epoxide hydrolase (sEH) and leukotriene A4 hydrolase (LTA4H), as well as the nuclear receptors farnesoid X receptor (FXR) and retinoid X receptor (RXR). For several combinations of these proteins, sEH/5‐LOX,^[11]^ sEH/LTA4H,^[12]^ and sEH/FXR,^[13]^ feasibility of a DML has been demonstrated. Despite their common feature of binding lipids and fatty acid mimetics,^[14]^ the protein fold, catalysed reaction, as well as binding site shape and residues strongly differ in all five targets. 5‐LOX is an iron‐dependent enzyme catalysing the epoxidation of arachidonic acid to leukotriene A4 (LTA4), which is subsequently converted to LTB4 by zinc‐dependent LTA4H.^[15]^ sEH, by converting fatty acid epoxides to their corresponding diols performs a different hydrolysis reaction of polyunsaturated fatty acid epoxides.^[16]^ FXR and RXR belong to the family of nuclear receptors and are activated by bile acids and fatty acids, respectively.^[17]^

In order to computationally pre‐evaluate the chemical ligand space of the protein targets and select a suitable fragment collection, we trained a self‐organizing map (SOM)^[18]^ on the known modulators of all five targets with an IC_50_ or *K*_i_ <10 μM retrieved from the ChemblDB^[19]^ v.24 as well as on the Prestwick Chemical library (Prestwick Chemical, Illkirch, France) containing off‐patent approved drugs. The FragFP descriptor, a substructure‐based fingerprint was calculated for all compounds using OSIRIS DataWarrior v.5.0.0 (www.openmolecules.org) and employed for training of a SOM with 50×50 neurons. Analysis of the SOM revealed that the active compound for the individual target proteins occupy distinct clusters (Figure 1), whereas the approved drugs were widely distributed between the clusters, suggesting that a fragment library derived from these compounds is suitable to discover actives on all five targets. Based on this observation, we selected the core set of the Prestwick Drug‐Fragment Library (PDFL) comprising 480 compounds for the fragment screening approach. This structurally diverse fragment library was generated by virtual fragmentation of approved drugs and provides a broad distribution of chemical motifs and functional groups present in bioactive compounds.

**Figure 1:**
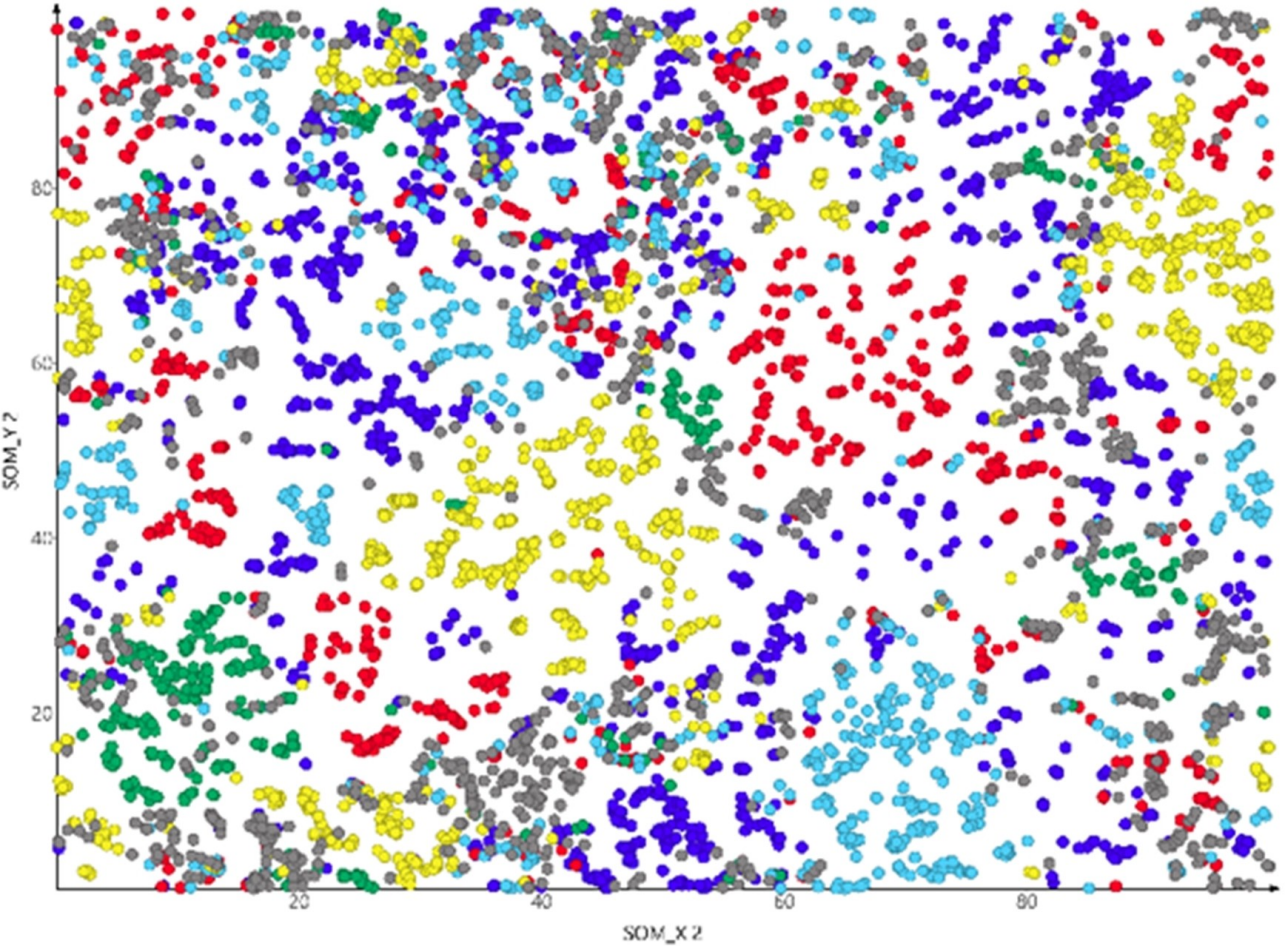
Self‐organizing map of the active compounds for 5‐LOX (blue), FXR (red), LTA4H (green), RXR (light blue), and sEH (yellow), as well as the Prestwick Chemical library of approved drugs (grey).

For the primary screen of this fragment library, we employed differential scanning fluorimetry (DSF, also known as thermal shift). DSF is generally applicable to a large panel of protein targets and provides a robust, low‐cost screening technology for fragment‐based approaches.^[20]^ All five proteins of our interest are soluble and were recombinantly expressed in *Escherichia coli*. We performed an initial fragment screen on all five targets by DSF. The use of a uniform screening method for all targets ensured consistent data and provided the opportunity to identify false positive hits which interfered with the screening technology. The conditions of the DSF assay were optimized for the desired screening and validated with reference compounds (Table S1 in the Supporting Information). Compounds causing a positive thermal shift Δ*T*_m_≥1.0 °C were considered as active. Of note, the reference 5‐LOX inhibitor did not exhibit protein stabilization observed by a thermal shift but the shape of the resulting melting curve was considered to be sufficient for screening and a lower cutoff (Δ*T*_m_≥0.9 °C) was applied for 5‐LOX.

DSF measurement of the PDFL (single concentration of 500 μM, duplicates, 96‐well format) on the five proteins retrieved 19 fragment hits on sEH, 28 on LTA4H, 12 on 5‐LOX, 16 on RXRα, and 14 on FXR, respectively. Melting point distributions of the screening library for all five targets are depicted in Figure 2. Amongst the hits, three compounds (Table S2) gave a positive signal on all five proteins likely qualifying them as pan assay interference compounds (PAINS).^[21]^ Fragments binding to multiple targets (Scheme 1) were identified for seven out of ten target combinations (Figure 2, Table 1). Among them, fragments **4**, **6**, **7**, and **9** match previously identified privileged scaffolds for fatty acid mimetics.^[14]^ Substructure search among the active compounds revealed that the 4‐benzylphenol **4** as well as 4‐hydroxybiphenyl **9** have been successfully incorporated in ligands of all five targets. Active ligands containing *N*‐phenylbenzamide **6** and the *N*‐phenylbenzylamine **7** have been described for four of the protein targets. Furthermore, indole, which has been characterized as a privileged heterocycle for fatty acid mimetics,^[14]^ is present in fragments **5** and **10**. Dual 5‐LOX/RXR ligand **1** was present in 5‐LOX inhibitors, while dual FXR/LTA4H modulator **3** is found among known FXR ligands. Fragments **2** (sEH/5‐LOX) and **8** (LTA4H/RXR) are novel scaffolds not appearing in known ligands of any of the five targets.

**Figure 2:**
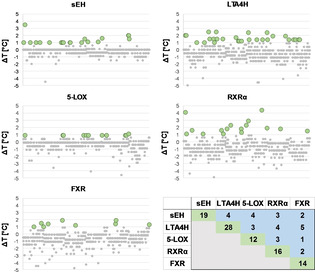
Melting‐point distributions for the fragment library screening on five target proteins obtained from DSF screening. Individual melting points represent the mean of duplicate measurements. Only compounds causing a positive thermal shift Δ*T*_m_≥1.0 °C (Δ*T*_m_≥0.9 °C for 5‐LOX) were considered further (green dots).

**Scheme 1:**
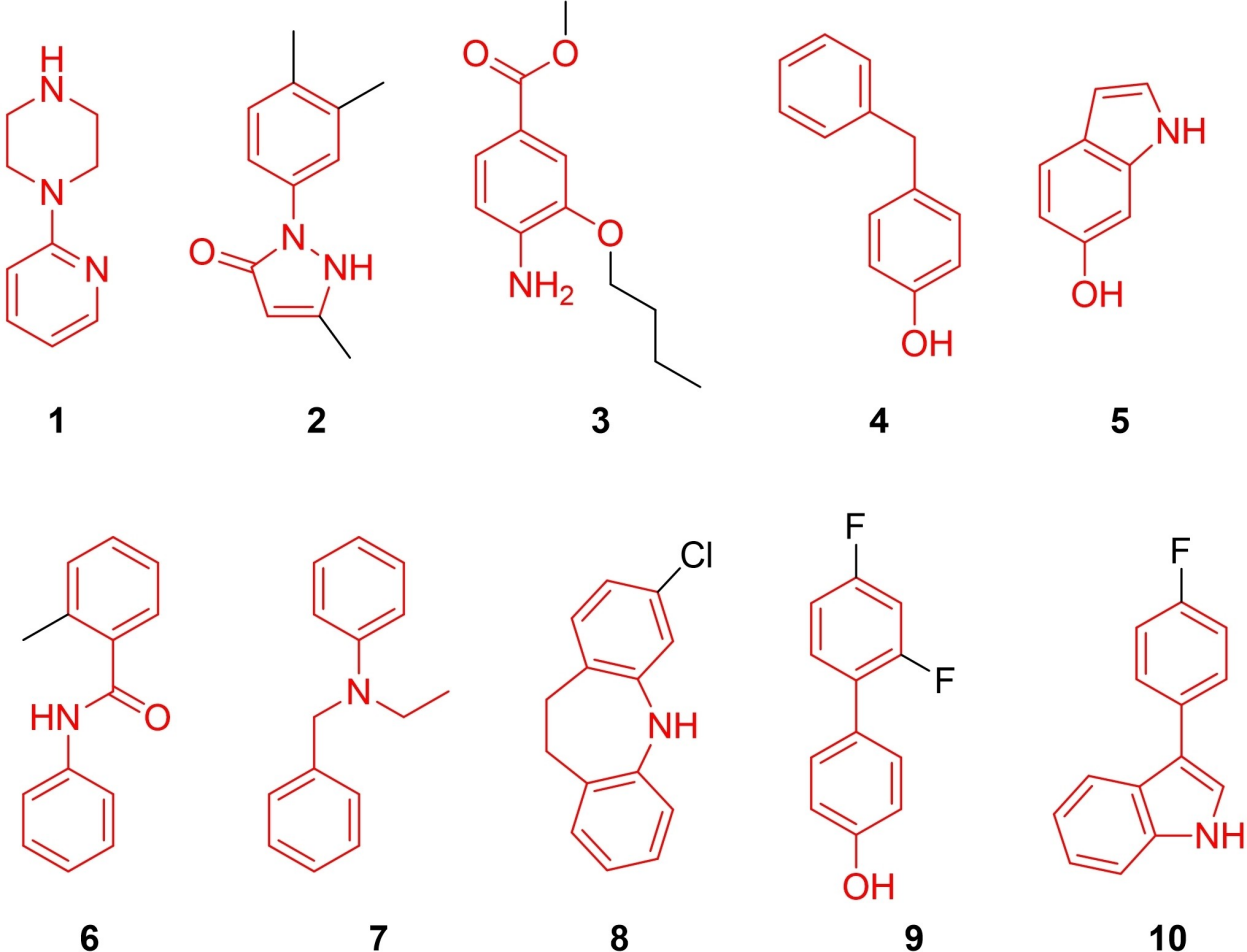
Validated hit compounds from the DSF screen displaying a thermal shift towards more than one target. Scaffolds are highlighted in red. Surprisingly, despite all five proteins binding fatty acid derivatives, no fragment hit contained a carboxylate moiety even though the screening library contained 47 (10 %) carboxylic acids. This can be explained by the fact that enthalpy‐driven binding of fatty‐acid mimetics results from occupation of hydrophobic subpockets rather than from mimicking the carboxylate interactions.^[14]^

**Table 1:** Hit compounds from the DSF screen causing a thermal shift for more than one target. DSF/Δ*T*_m_ is reported in mean [°C].

	sEH	LTA4H	5‐LOX	RXRα	FXR
**1**	0.0±0.0	−0.5±0.0	0.9±0.0	1.1±0.0	−0.1±0.0
**2**	2.2±0.0	−0.1±2.1	1.1±0.0	−9.5±24.0	0.3±0.0
**3**	0.0±0.0	2.5±0.0	−1.1±0.0	−2.9±0.0	1.4±0.7
**4**	0.0±0.0	1.5±0.0	−1.1±0.0	−2.4±0.7	5.4±0.7
**5**	0.0±0.0	1.7±0.0	−0.1±0.0	0.8±0.7	1.2±0.0
**6**	2.0±0.0	1.4±1.4	−1.4±2.1	−1.0±0.7	−1.2±0.7
**7**	1.0±0.0	0.5±0.0	0.9±0.0	−0.4±0.7	−0.6±0.7
**8**	0.0±0.0	1.5±0.0	−0.1±0.0	0.1±0.0	1.9±0.0
**9**	1.0±0.0	0.7±0.0	−1.1±0.0	2.3±0.0	21.7±3.5 ^[a]^
**10**	0.6±0.7	2.4±0.0	0.9±0.0	1.9±0.0	−11.6±2.1

[a] The melting curve is shown in the Supporting Information

For hit validation, we employed secondary assays with orthogonal readouts to confirm fragment activities. Biochemical activity assays were chosen for the enzymes sEH, LTA4H, and 5‐LOX. Compound activity on sEH and LTA4H was observed using recombinant protein and fluorogenic substrates, while activity on recombinant 5‐LOX was determined by HPLC‐based detection of product formation (5‐HETE). RXRα and FXR modulation was assessed in cell‐based hybrid reporter gene assays. All ten compounds were tested at a single concentration of 500 μM in the sEH and LTA4H activity assays and with a concentration of 100 μM on 5‐LOX, RXRα, and FXR. Fragments showing an inhibition greater than 50 % (sEH‐H and LTA4H) or greater than 70 % (5‐LOX) as well as the hits determined in the DSF experiments were further characterized in full concentration‐response curves between 0.1 and 1000 μM. Toxicity at concentrations of 100 μM and above in the cell‐based reporter gene assays prevented reliable characterization on FXR and RXRα, however. As alternative cell‐free assay we employed isothermal titration calorimetry (ITC) to determine binding affinity of the fragments towards the LBDs of both nuclear receptors. ITC was performed with a protein concentration of 50 μM and compound concentrations of 250 μM. Fragment activities in the secondary assays are reported in Table 2. DSF hits **2** (sEH/5‐LOX), **4** (LTA4H/FXR), **9** (sEH/FXR), and **10** (5‐LOX/LTA4H) were confirmed active in the orthogonal experiments. Ligand efficiency (LE) calculated from the respective IC_50_ or *K*_d_ values was found favorable for the confirmed hits (LE≥0.3 kcal/mol) rendering them as valuable starting points for multitarget design.^[22]^

**Table 2:** Validation of hit compounds.

	sEH				LTA4H				5‐LOX			RXRα			FXR		

Cmpd	IC_50_ [μM]	LE [kcal/mol]	*K*_d_ [μM]	scaffold found in bioactives	IC_50_ [μM]	LE [kcal/mol]	*K*_d_ [μM]	found in bioactives	IC_50_ [μM]	LE [kcal/mol]	found in bioactives	*K*_d_ [μM]	LE [kcal/mol]	found in bioactives	*K*_d_ [μM]	LE [kcal/mol]	found in bioactives
1	–	–	–	x	–	–	–		n.d.	–	x	n.b.	–		n.b.	–	
2	135.3±9.1	0.35	–		–	–	–		21.2±7.3	0.42		–	–		–	–	
3	>300	<0.3	–		24.6±3.2	0.39	–		–	–		‐	–		–	–	x
4	150.8±5.8	0.37	–	x	3.0±0.5	0.54	–	x	–	–	x	–	–	x	0.68	0.60	x
5	–	–	–		>300	0.39	–		19.9±2.4	0.64	x	–	–		n.b.	–	x
6	225.7±18.5	0.31	–	x	n.d.	–	–		–	–	x	–	–	x	–	–	x
7	56.6±5.4	0.36	–	x	>300	<0.29	–		118.0±45.1	0.33	x	–	–	x	–	–	x
8	140.7±34.1	0.33	–		n.d.	–	–		6.5±2.2	0.44		–	–		w.b.	–	
9	107.6±11.0	0.36	3.5	x	36.2±7.6	0.40	5.3	x	64.6±26.4	0.38	x	n.b.	–	x	b.	–	x
10	119.7±22.4	0.33	0.86		254.5±24.9	0.31	1.7		54.3±19.7	0.36		n.b.	–	x	n.b.	–

IC_50_ is reported in [μM] and represents the mean±standard deviation of three independent experiments, *K*_d_ is reported in [μM], and LE is reported in [kcal/mol]. n.d.: not determinable, n.b.: no binding, w.b.: weak binding.

In a previous study, we demonstrated that potent multitarget ligands can be identified among approved drugs and subsequently optimized using the SOSA approach.^[23]^ As mentioned before, PDFL compounds are derived from the Prestwick Chemical library. Thus, we performed a substructure search with each hit fragment in the Prestwick Chemical library and retrieved eight approved drugs (**11**–**18**) that contained a motif of a multitarget active fragment from the screening (Scheme 2). Drugs **11**–**18** were evaluated in the enzyme activity assays for sEH, LTA4H, and 5‐LOX as well as in the cellular reporter gene assays for FXR and RXR. None of the approved drugs 11–20 exhibited dual target activity, and their potencies were less favorable considering their molecular weights being higher than those of the validated fragment hits. Most interestingly, calcium channel blocker **15** (bepridil), a derivative of fragment **7**, inhibited 5‐LOX with an IC_50_ of 3.2 μM, while completely loosing activity towards sEH. Thus, simple expansion of drug‐derived fragment hits to the respective drugs is not sufficient to discover leads for multitarget design.

**Scheme 2:**
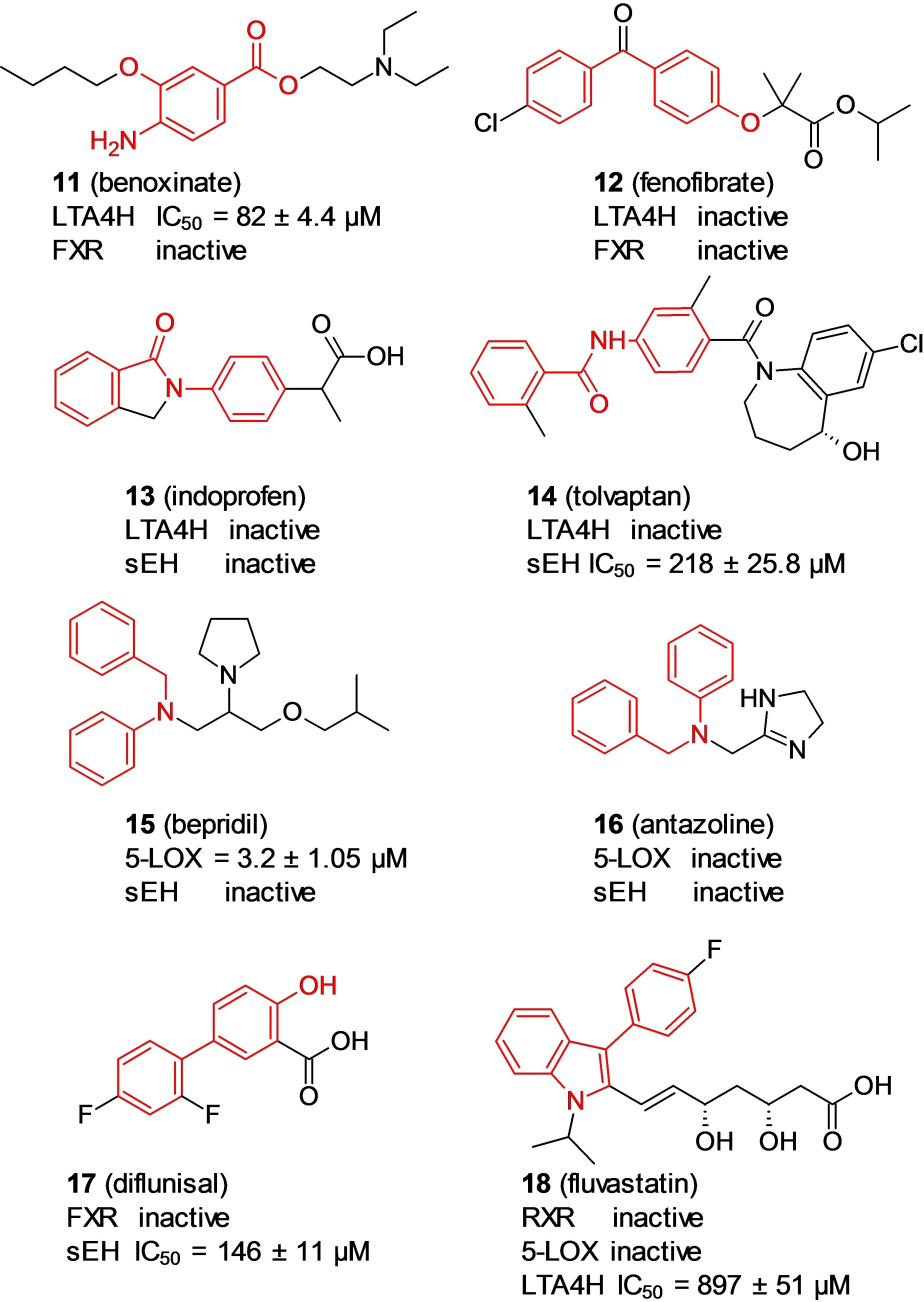
Approved drugs from Prestwick Chemical library containing scaffolds of identified dual target fragments and their activity towards targets previously addressed by the parent fragments.

The systematic fashion of our approach also allows for comparison to previous attempts to identify dual ligands by means of rational design of a starting fragment for subsequent optimization (Scheme 3). The biphenyl fragment **9** was identified as a dual sEH/FXR ligand. Substructure search of ChemblDB revealed that the biphenyl fragment was previously incorporated in potent FXR partial agonist **19** ^[24]^ and sEH inhibitor **20**.^[25]^ Rationally designed dual fragment **21**,^[13]^ which enabled development of potent dual sEH/FXR modulators, was not present in the screening library. The most similar (Tanimoto coefficient on FragFP) fragment **22** displayed no activity in the DSF screen. The same holds true for the dual 5‐LOX/sEH fragment inhibitor **23**, which was identified previously by virtual screening,^[26]^ while its closest neighbor **24** displayed no activity. This observation leads to the assumption that a fragment‐based screening approach is complementary to rational design of multitarget ligands and vice versa.

**Scheme 3:**
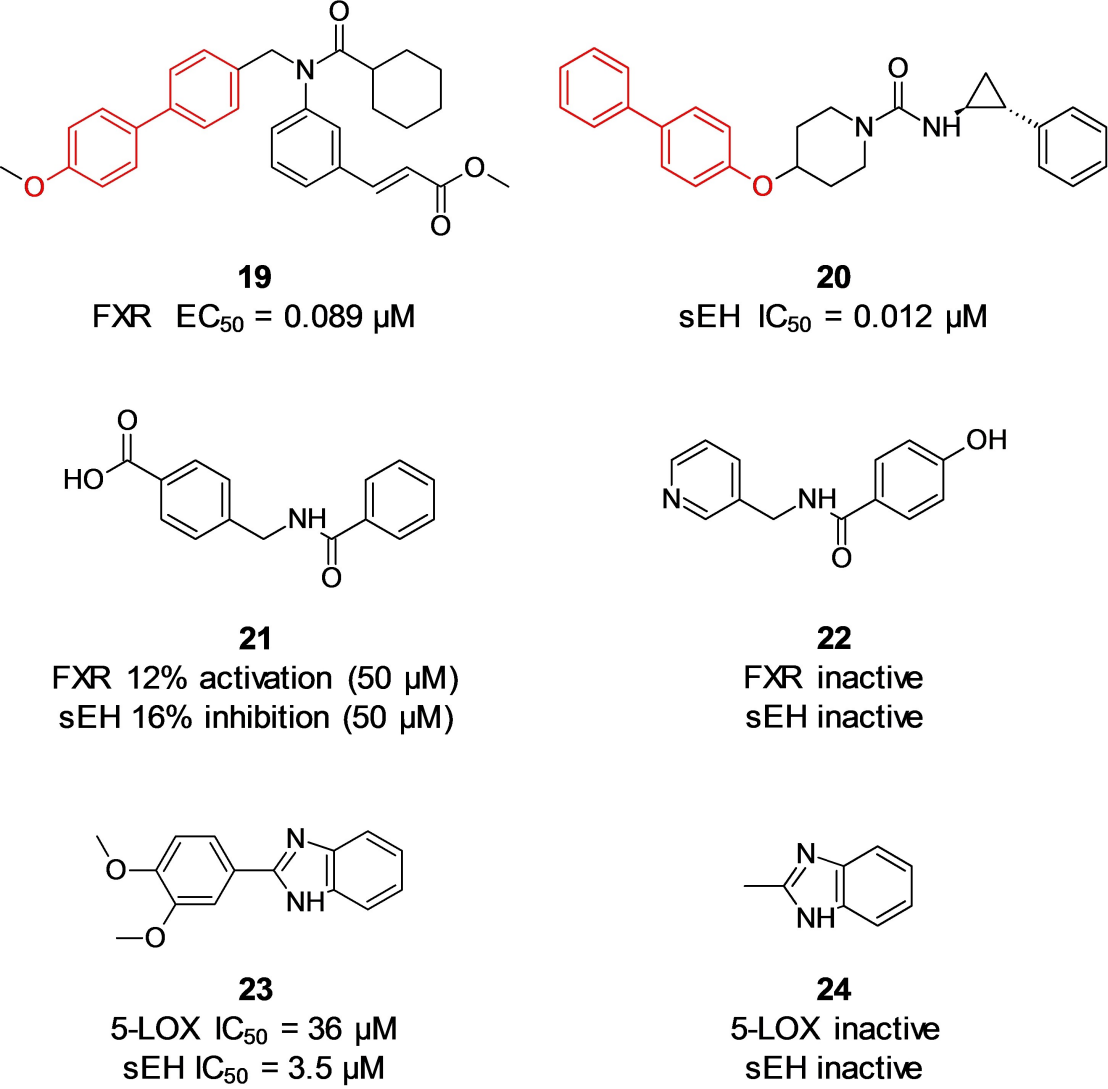
Previously identified ligands and their closest neighbors in the PDFL.

Recapitulating our observations in this systematic model study, several lessons for multitarget drug design can be deduced:

**Fragment‐based approaches can be considered as an option for multitarget drug design**

For six out of ten target combinations, fragment hits were successfully obtained from library screening, and for four out of ten, the hits could be validated in an orthogonal assay system. Most previous multitarget‐optimization studies have relied on a rational combination of the pharmacophores. This approach is in many cases highly efficient, yet, it requires deep understanding of the pharmacophores and the underlying structure–activity relationship for the targets of interest, which is not always available. Furthermore, linking, merging, or fusing pharmacophores mostly yields previously described chemical scaffolds or combinations thereof, whereas a systematic fragment‐based approach is unbiased by previously described ligands.[^5^, ^27^]

**The size of the compounds matters**

This systematic experimental fragment screening fully supports the theoretical assumptions by Hann et al.^[9]^ and Hopkins et al.^[10]^ – the target promiscuity of chemical compounds decreases with increasing molecular weight. While the fragments **1**–**10** were able to hit multiple targets, their larger approved drug counterparts **11**–**18** exhibited only weak selective activity with respect to their molecular weight. Therefore, a promising starting point for a multitarget ligand discovery campaign is a low‐molecular‐weight hit, exactly as for the discovery of single‐target drugs.

**The size and the composition of the library matters**

The hit rates for fragment‐based screening are generally described in the range of 2–8 %.[^28^, ^29^] In this study, hit rates of 2.5–5.8 % were reached on the individual target. Furthermore, six out of ten dual target hits represent privileged scaffolds for fatty acid mimetics. The concept of using focused fragment libraries was successfully applied for kinases^[30]^ or metal‐binding proteins^[31]^ and could be potentially adopted for multitarget fragment screening.

**The screening technology matters**

Several studies have evaluated the applicability of different screening technologies for fragment identification. In this study, DSF screening was employed as a universal low cost method for primary screening. However, follow‐up characterization of the fragments using fluorescence‐based activity assays for sEH and LTA4H revealed that even compounds without a positive shift in DSF can exhibit inhibitory activity, which should certainly be handled with care. Furthermore, given the low probability to identify a multitarget fragment hit, a more permissive primary screening technique would offer advantages. Screening by X‐ray crystallography^[32]^ or NMR spectroscopy^[33]^ would additionally provide valuable structural information which is indispensable for fragment‐based approaches, especially in the design of multitarget compounds.

Given the topicality of multitarget drug discovery, fragment‐based design offers a yet underexplored possibility to identify starting points and pave the way to DMLs with favorable properties.

## Conflict of interest

The authors declare no conflict of interest.

## Supporting information

As a service to our authors and readers, this journal provides supporting information supplied by the authors. Such materials are peer reviewed and may be re‐organized for online delivery, but are not copy‐edited or typeset. Technical support issues arising from supporting information (other than missing files) should be addressed to the authors.

Supplementary
