# Supplementary material for: Systematic Assessment of Fragment Identification for Multitarget Drug Design
Source: ChemMedChem. 2021 Feb 4;16(7):1088–92. doi: 10.1002/cmdc.202000858 (PMC8049054; doi:10.1002/cmdc.202000858)
Supplement: Supplementary file 1 — Supplementary [file CMDC-16-1088-s001.pdf]

ChemMedChem

Supporting Information

Systematic Assessment of Fragment Identification for Multitarget Drug Design

Steffen Brunst, Jan S. Kramer, Whitney Kilu, Jan Heering, Julius Pollinger, Kerstin Hiesinger, Sven George, Dieter Steinhilber, Daniel Merk, and Ewgenij Proschak*

Supporting Information

Supplementary Tables 2
General 3
SOM training 3
Cloning of FXR and RXR constructs 3
FXR cloning: 3
RXRα cloning: 4
Protein expression and purification 5
FXR, LTA4H, sEH-H and sEH-Fl: 5
5-LOX: 5
RXRα: 6
DSF screening 7
Orthogonal assay systems 8
Fragments: 8
Approved drugs: 8
sEH-H activity assay: 8
LTA4H activity assay: 8
5-LOX activity assay: 8
Hybrid reporter gene assay FXR and RXRα: 8
ITC measurements for sEH-H, LTA4H, FXR, and RXRα: 8
Results of the fragment testing 9
DSF experiments: 9
sEH-H: 9
LTA4H: 9
5-LOX: 10
RXRα: 10
FXR: 10
IC_{50} measurement results: 11
sEH-H activity: 11
LTA4H activity: 11
5-LOX activity: 12
ITC Measurements results: 12
sEH-H measurement: 12
LTA4H measurement: 14

FXR measurement: 16
Results of the tested approved drugs 19
sEH-H activity: 19
LTA4H activity: 19
5-LOX activity: 19
SMILES Codes of Prestwick Drug-Fragment Library (PDFL) 20
References 26

Supplementary Tables

Table S1: DSF validated with reference compounds. Assay conditions according to fragment testing. sEH-H, LTA4H and FXR were measured as triplicates and repeated 3 times. 5-LOX and RXRα were measured once as triplicate.

Protein	Reference compound (ref)	T_M(DMSO) [°C]	T_M(ref) [°C]	ΔT_M
sEH-H	50 µM CIU	56.7 ± 0.5	62.2 ± 0.4	5.6 ± 0.5
LTA4H	50 µM bestatin	54.8 ± 0.4	60.7 ± 0.9	5.9 ± 0.7
5-LOX	100 µM atreleuton	64.0 ± 0.0	63.7 ± 0.6	n.d.
RXRα	50 µM SR11237	No melting curve	59.0 ± 0.0	n.d.
FXR	60 µM GW4064	44.9 ± 0.6	n.d.	n.d.

Table S2: Excluded Pan Assay Interference Compounds (PAINS).

Structure	Reason for exclusion
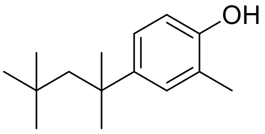	Shift higher than the threshold on all five proteins.
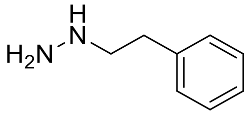	Shift higher than the threshold for sEH-H, LTA4H, and RXR. Contains reactive hydrazine functional group
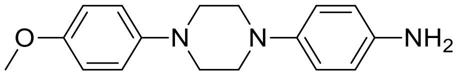	PAIN structure determined by KNIME (Version 4. 1.2) using the "RDKit Molecule Catalogue Filter" node[1].

General

The Prestwick Drug-Fragment Library and all compounds tested in the orthogonal assay systems were purchased from Prestwick Chemical Librarys (Illkirch, France). The compounds measured in the ITC experiments were purchased from TCI Deutschland GmbH (compound 1), Fluorochem Ltd (compound 4, 5 and 8) and BLD Pharmatech GmbH (compound 9 and 10). The restriction enzymes BamHI and XhoI were purchased from New England Biolabs GmbH. LB Broth and SYPRO® Orange Protein Gel Stain were purchased from Invitrogen™. If not otherwise declared the used chemicals were either purchased from AppliChem GmbH, Thermo Fisher (Kandel) GmbH, Merck KGaA or Carl Roth GmbH + Co. KG.

Purity of all active compounds was determined with a LCMS 2020 and a coupled UV detector module SPD-20A from Shimadzu. The columns Luna 10u C18(2) 100A (250 x 4.60 mm) and Luna 10u CN 100A (250 x 4.60 mm) from Phenomenex were used with a flow rate of 1 mL/min. The eluents were 0.1% formic acid and acetonitrile and the detection occurred via UV absorption at two wavelengths (254 and 280 nm). All final compounds exhibit a purity over 95% at 254 nm and 280 nm. The methods were as followed:

Method A: linear gradient from 40% to 90% acetonitrile within 10 min, 90% acetonitrile were hold for another 6 min, linear gradient from 90% to 40% within 2 min, 40% acetonitrile were hold for 2 min

Method B: hold 5 % acetonitrile for 2 min, linear gradient from 5% to 90% acetonitrile within 12 min, 90% acetonitrile for further 4 min, linear gradient from 90% to 5% within 1 min, 90% acetonitrile were hold for 1 min

Method C: linear gradient from 30% to 90% acetonitrile within 10 min, 90% acetonitrile were hold for another 5 min, linear gradient from 90% to 30% within 1 min, 30% acetonitrile were hold for 4 min

SOM training

The Kohonen network was used only for visualization of the chemical space. Overall, 16,791 compounds (1,280 approved drugs, 2,630 sEH inhibitors, 5,163 5-LO inhibitors, 1,118 LTA4H inhibitors, 2,831 RXR ligands, 3,769 FXR ligands) were used for training. Approved drugs were retrieved from the Prestwick Drug Library, while the other compounds were downloaded from ChemblDB v24 using the human target name as search item via the DataWarrior "Search Chembl Database" interface. FragFP fingerprints were calculated and a 50x50 Kohonen network was trained using default settings.

Cloning of FXR and RXR constructs

FXR cloning:

The FXR LBD construct (aa244-472) was cloned using the pFA-CMV-hFXR-LBD[2] as PCR template (forward primer 5'-CCCCGGATCCGAACTGACCCCAGATCAACAG-3' and reverse primer 5'-CCCCCTCGAGTCATTATCACTGCACGTCCCAGATTTC-3). The PCR product as well as a modified pET29-vector were linearized with the restriction enzymes BamHI and XhoI and purified using NucleoSpin® Gel and PCR Clean-up (MACHERY-NAGEL) according to supplier protocol. The purified products were ligated using T4-ligase for 1h at 37 °C. The mixture was then used to transform E. *coli* (DH5α) by standard heat shock transformation protocol. Single clones were selected from agarose plates, containing a final concentration of 100 µg/ml Kanamycin and multiplied plasmids were extracted using NucleoSpin® Plasmid EasyPure (MACHERY-NAGEL) according to supplier protocol. The sequence of the insert was verified by Sanger sequencing. (Microsynth Seqlab) .The final construct carried an N-terminal His_6-tag and a TEV site followed by the FXR LBD.

DNA sequence of His-TEV-FXR(aa2244-472) construct:
ATGGGCCATCACCATCACCATCACGACTACGACATCCCGACTACCGAAAACCTGTACTTCCAGGGATCCGAACTGACCCCA
GATCAACAGACTCTTCTACATTTTATTATGGATTCATATAACAAACAGAGGATGCCTCAGGAAATAACAAATAAAATTTTAAAA
GAAGAATTCAGTGCAGAAGAAAATTTTCTCATTTTGACGGAAATGGCAACCAATCATGTACAGGTTCTTGTAGAATTCACAAA
AAAGCTACCAGGATTTCAGACTTTGGACCATGAAGACCAGATTGCTTTGCTGAAAGGGTCTGCGGTTGAAGCTATGTTCCTT
CGTTCAGCTGAGATTTTCAATAAGAAACTTCCGTCTGGGCATTCTGACCTATTGGAAGAAAGAATTCGAAATAGTGGTATCT
CTGATGAATATATAACACCTATGTTTAGTTTTTATAAAAGTATTGGGGAACTGAAAATGACTCAAGAGGAGTATGCTCTGCTT
ACAGCAATTGTTATCCTGTCTCCAGATAGACAATACATAAAGGATAGAGAGGCAGTAGAGAAGCTTCAGGAGCCACTTCTTG
ATGTGCTACAAAAGTTGTGTAAGATTCACCAGCCTGAAAATCCTCAACACTTTGCCTGTCTCCTGGGTCGCCTGACTGAATT
ACGGACATTCAATCATCACCACGCTGAGATGCTGATGTCATGGAGAGTAAACGACCACAAGTTTACCCCACTTCTCTGTGAA
ATCTGGGACGTGCAGTGA

Protein sequence of His-TEV-FXR(aa2244-472) construct:
MGHHHHHHDYDIPTTENLYFQGSELTPDQQTLLHFIMDSYNKQRMPQEITNKILKEEFSAEENFLILTEMATNHVQVLVEFTKKLP
GFQTLDHEDQIALLKGSAVEAMFLRSAEIFNKKLPSGHSDLLEERIRNSGISDEYITPMFSFYKSIGELKMTQEEYALLTAIVILSPDR
QYIKDREAVEKLQEPLLDVLQKLCKIHQPENPQHFACLLGRLTELRTFNHHHAEMLMSWRVNDHKFTPLLCEIWDVQ

Sequencing Data:

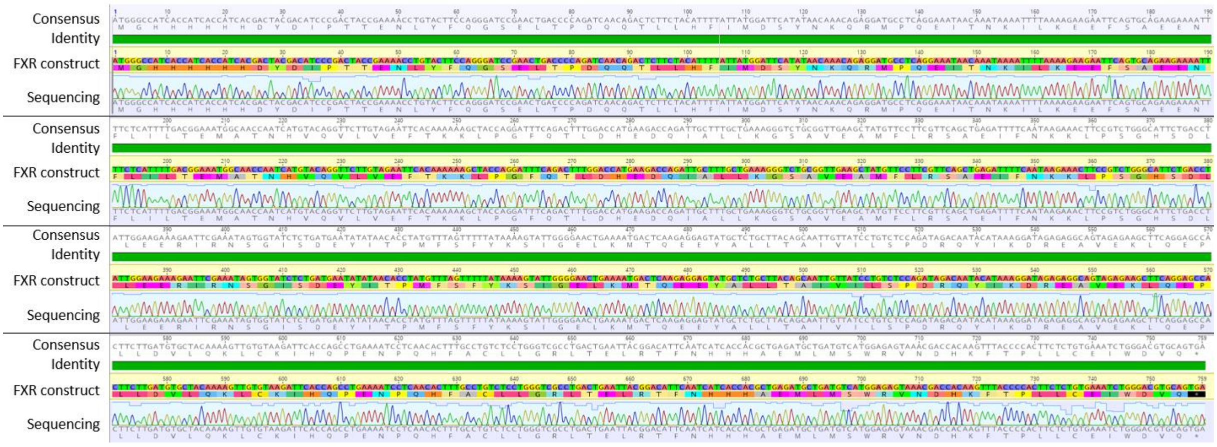

Figure S1: Alignment of the His-TEV-FXR(aa244-472) construct and the sequencing data (T7term primer (TGCTAGTTATTGCTCAGCGG) from Microsynth Seqlab) using the standard settings of "map to reference" with the software Geneious 11.0.5.

RXRα cloning:
The wildtype RXRα LBD construct (aa225-462) was first amplified via PCR (forward primer 5'-CCGAAAACCTGTACTTCCAGGGATCCACCTCTTCCGCCAATGAAGATATGCCGG-3' and reverse primer 5'-CTGCAGGTCGACCTCGAGTCATTAGGTCATCTGATGCGGAGCTTCC-3') from a tag free RXRα LBD construct and carried an N-terminal TEV recognition and cleavage side. The PCR product was again cloned into a pMAL vector with an ampicillin resistance using PCR (forward primer 5'-ATGACTCGAGGTCGACCTGCAGGCAAGCTTGG-3' and reverse primer 5'-CTGCAGGTCGACCTCGAGTCATTAGGTCATCTGATGCGGAGCTTCC-3'). The final construct carried an N-terminal maltose binding protein (MBP) tag followed by a poly-N-tag, a His_8-tag and a TEV site.

DNA sequence of MBP-His-TEV-RXR(aa225-462) construct:
ATGAAAACTGAAGAAGGTAAACTGGTAATCTGGATTAACGGCGATAAAGGCTATAACGGTCTCGCTGAAGTCGGTAAGAAA
TTCGAGAAAGATACCGGAATTAAAGTCACCGTTGAGCATCCGGATAAACTGGAAGAGAAATTCCCACAGGTTGCGGCAACT
GGCGATGGCCCTGACATTATCTTCTGGGCACACGACCGCTTTGGTGGCTACGCTCAATCTGGCCTGTTGGCTGAAATCACC
CCGGACAAAGCGTTCCAGGACAAGCTGTATCCGTTTACCTGGGATGCCGTACGTTACAACGGCAAGCTGATTGCTTACCCG
ATCGCTGTTGAAGCGTTATCGCTGATTTATAACAAAGATCTGCTGCCGAACCCGCCAAAAACCTGGGAAGAGATCCCGGCG
CTGGATAAAGAACTGAAAGCGAAAGGTAAGAGCGCGCTGATGTTCAACCTGCAAGAACCGTACTTCACCTGGCCGCTGATT
GCTGCTGACGGGGGTTATGCGTTCAAGTATGAAAACGGCAAGTACGACATTAAAGACGTGGGCGTGGATAACGCTGGCGC
GAAAGCGGGTCTGACCTTCCTGGTTGACCTGATTAAAAACAAACACATGAATGCAGACACCGATTACTCCATCGCAGAAGC
TGCCTTTAATAAAGGCGAAACAGCGATGACCATCAACGGCCCGTGGGCATGGTCCAACATCGACACCAGCAAAGTGAATTA
TGGTGTAACGGTACTGCCGACCTTCAAGGGTCAACCATCCAAACCGTTCGTTGGCGTGCTGAGCGCAGGTATTAACGCCG
CCAGTCCGAACAAAGAGCTGGCAAAAGAGTTCCTCGAAAACTATCTGCTGACTGATGAAGGTCTGGAAGCGGTTAATAAAG
ACAAACCGCTGGGTGCCGTAGCGCTGAAGTCTTACGAGGAAGAGTTGGCGAAAGATCCACGTATTGCCGCCACCATGGAA
AACGCCCAGAAAGGTGAAATCATGCCGAACATCCCGCAGATGTCCGCTTTCTGGTATGCCGTGCGTACTGCGGTGATCAAC
GCCGCCAGCGGTCGTCAGACTGTCGATGAAGCCCTGAAAGACGCGCAGACTAATTCGAGCTCGAACAACAACAACAATAA
CAATAACAACAACCTCGGGATCGAGGGTACCCATCACCATCACCATCACCATCACCCGGGTACCGAAAACCTGTACTTCCA
GGGATCCACCTCTTCCGCCAATGAAGATATGCCGGTTGAACGTATTCTGGAAGCAGAACTGGCAGTTGAACCGAAAACCGA
AACCTATGTTGAAGCAAATATGGGTCTGAATCCGAGCAGCCCGAATGATCCGGTTACCAATATTTGTCAGGCAGCAGATAAA
CAACTGTTTACCCTGGTTGAATGGGCAAAACGTATTCCGCATTTTAGCGAACTGCCGCTGGATGATCAGGTTATTCTGCTGC
GTGCAGGTTGGAATGAACTGCTGATTGCCAGCTTTAGCCATCGTAGCATTGCAGTTAAAGATGGCATTCTGCTGGCAACCG
GTCTGCATGTTCATCGTAATAGCGCACATAGTGCCGGTGTTGGTGCAATTTTTGATCGTGTTCTGACCGAACTGGTTAGCAA
AATGCGTGATATGCAGATGGATAAAACAGAACTGGGTTGTCTGCGTGCAATTGTGCTGTTTAATCCGGATAGCAAAGGTCT
GAGCAATCCTGCCGAAGTTGAAGCACTGCGTGAAAAAGTTTATGCAAGCCTGGAAGCCTATTGCAAACATAAATATCCGGA
ACAGCCTGGTCGTTTTGCCAAACTGCTGCTGCGTCTGCCTGCACTGCGTAGTATTGGTCTGAAATGTCTGGAACACCTGTT
TTTTTTCAAACTGATTGGTGATACCCCGATCGATACCTTTCTGATGGAAATGCTGGAAGCTCCGCATCAGATGACCTAA

Protein sequence of MBP-His-TEV-RXR(aa225-462) construct:
MKTEEGKLVIWINGDKGYNGLAEVGKKFEKDTGIKVTVEHPDKLEEKFPQVAATGDGPDIIFWAHDRFGGYAQSGLLAEITPDKA
FQDKLYPFTWDAVRYNGKLIAYPIAVEALSLIYNKDLLPNPPKTWEEIPALDKELKAKGKSALMFNLQEPYFTWPLIAADGGYAFKY
ENGKYDIKDVGVDNAGAKAGLTFLVDLIKNKHMNADTDYSIAEAAFNKGETAMTINGPWAWSNIDTSKVNYGVTVLPTFKGQPSK
PFVGVLSAGINAASPNKELAKEFLENYLLTDEGLEAVNKDKPLGAVALKSYEEELAKDPRIAATMENAQKGEIMPNIPQMSAFWYA
VRTAVINAASGRQTVDEALKDAQTNSSSNNNNNNNNNNLGIEGTHHHHHHHHPGTENLYFQGSTSSANEDMPVERILEAELAVE
PKTETYVEANMGLNPSSPNDPVTNICQAADKQLFTLVEWAKRIPHFSELPLDDQVILLRAGWNELLIASFSHRSIAVKDGILLATGL
HVHRNSAHSAGVGAIFDRVLTELVSKMRDMQMDKTELGCLRAIVLFNPDSKGLSNPAEVEALREKVYASLEAYCKHKYPEQPGR
FAKLLLRLPALRSIGLKCLEHLFFFKLIGDTPIDTFLMEMLEAPHQMT

Sequencing Data:

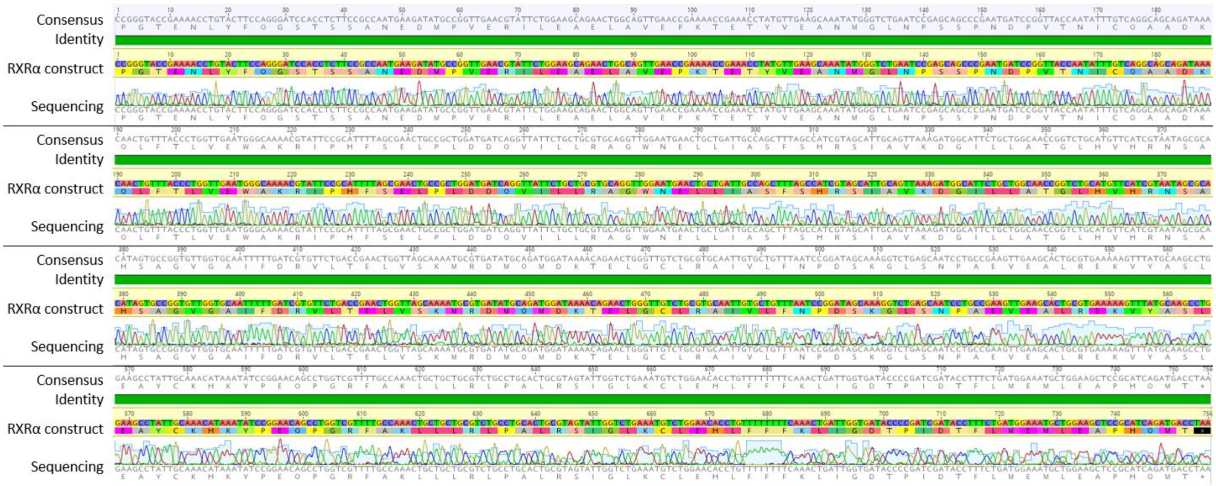

Figure S2: Alignment of the MBP-His-TEV-RXR(aa225-462) construct and the sequencing data starting at the end of the MBP (MalE primer (GGTCGTCAGACTGTCGATG) from Microsynth Seqlab) using the standard settings of "map to reference" with the software Geneious 11.0.5.

Protein expression and purification

FXR, LTA4H, sEH-H and sEH-Fl:

The LTA4H was purified as previously described[3] and FXR was purified adapted from Merk et. al.[4]. In brief, for the expression of LTA4H and FXR transformed *E.coli BL21 (DE3)* cells were grown in LB Broth supplemented with 100 µg/ml Kanamycin at a temperature of 37 °C and shaking speed of 180 rpm until an OD_{600} of 0.8 was reached. Then the cultures were induced by the addition of isopropyl β-D-thiogalactopyranosid (IPTG), with a final concentration of 400 µM in case of the LTA4H and 100 µM for FXR. After induction the incubation temperature was lowered to 21 °C for LTA4H and 18 °C in case of FXR. Cultures were harvested after ~ 18 h by centrifugation at 4 °C with 5500 x g for 20 min (centrifuge: Sorvall LYNX 4000 super speed centrifuge; rotor: F12-6X500-LEX fiberlite). For the expression of sEH-H and sEH-Fl transformed *E.coli BL21 (DE3)* cells were grown in 5052 media supplemented with 100 µg/ml kanamycin at 37 °C and 180 rpm as described by Hahn et al.[5] and Lukin et al.[6]. Temperature was reduced to 16 °C after 3 h and cells were harvested by centrifugation after additional 42 h of incubation. The cell pellets were either stored at -20 °C or directly suspended in buffer A supplemented with DNAse I and protease inhibitor mix (cOmplete™, EDTA-free Protease Inhibitor Cocktail, Roche). Cell suspension was lysed by a cell homogenizer (Invensys APV-1000 Homogenizer, Denmark). The lysed cells were centrifuged with 43992 x g at 4 °C for 1 h (centrifuge: Sorval RC 5B Plus; rotor: HFA22.50). The supernatant was filtrated using a 0.45 mm syringe filter, before the filtrate was further purified by using an ÄKTA purifier (GE Healthcare) with a 5 ml HisTrap HP (GE Healthcare) equilibrated and run in buffer A. The HisTrap was eluted using buffer B and the protein concentration of the elution fractions was measured. The fractions containing protein were pooled and if necessary the volume was reduced to 5 ml by using an Amicon® Ultra-15 Centrifugal Filter Unit with a 3 kDa cutoff and centrifuging at 4°C with 2500 x g (centrifuge: 5810R; rotor: F-34-6-38). In case of FXR, LTA4H and sEH-H the protein was filtrated through a 0.22 mm filter and the 5 ml were further purified by using the ÄKTA purifier (GE Healthcare) running in buffer C and a HiLoad 16/600 Superdex 75 pg™ (GE Healthcare) for FXR and a HiLoad 16/600 Superdex 200 pg™ (GE Healthcare) in case of LTA4H and sEH-H. sEH-Fl was instead dialyzed two times against ~ 100x volume of buffer C for about 8h each. The purity of the protein was evaluated by SDS page using a 14% polyacrylamide gel followed by Coomassi staining. The amount of protein was determined by measuring the sample with a NanoDrop™ 2000c. The fractions containing pure protein were pooled. They were either used for ITC experiments or in case of sEH-H and sEH-Fl the pooled fractions were supplemented with glycerol to a final concentration of 20%. The pooled fractions were then frozen in liquid nitrogen and then stored at -80 °C.

For the ITC experiments FXR was expressed as described above but after the purification using a 5 ml HisTrap HP (GE Healthcare) the fractions containing the protein were pooled and a self-produced TEV protease containing a 6xHis tag was added with a molar ratio of 1:10. The mixture was dialyzed overnight using buffer A and a dialysis membrane with a 3 kDa MWCO. Then a second run using a 5 ml HisTrap HP (GE Healthcare) was performed. Proteins without His-tag were in the flow through. These fractions were concentrated using an Amicon® Ultra-15 Centrifugal Filter Unit with a 10 kDa cutoff centrifuged at 4 °C with 2500 x g (centrifuge: 5810R; rotor: F-34-6-38) until a final volume of 5 ml was reached. Concentrated sample was further purified by gel filtration in an analog fashion to the normal FXR purification. From the fractions containing the highest amount of protein an SDS page was performed and the purest fractions were pooled and dialyzed two times for 12h in 1 L buffer C. The protein was frozen in liquid nitrogen and stored at -80 °C. The dialysis buffer was stored at -20 °C.

5-LOX:

The 5-LOX protein was expressed and purified as previously published[7,8]. In short the transformed *E.coli BL21 (DE3)* cells were grown in LB Broth supplemented with 50 µg/ml ampicillin at a temperature of 37 °C and shaking speed of 180 rpm for 5 h. Then the temperature was reduced to 21 °C and the cultures were induced by adding IPTG to a final concentration of 200 µM. After growing overnight, the cells were harvested by centrifugation (centrifuge: Sorval RC 5B Plus; rotor: HFA12.500). The cells were

suspended using 20 ml buffer A per Liter of expression culture and chilled for 20 min on ice. The suspended cells were lysed by sonification (Bandolin MS73) 3x 1 min with 90% intensity. Cell homogenate was centrifuged (centrifuge: Sorval® RC-5B Refrigerated Superspeed Centrifuge; rotor: 5534) for 15 min at 4 °C with 10,000 x g, before the supernatant was further centrifuged for 70 min at 4 °C with 100,000 x g (centrifuge: Optima™ LE-80K Ultracentrifuge; rotor: Beckman fixed angle rotor 55.2 Ti). The supernatant was applied on a custom-made ATP-column with a column volume of ~ 3.5 ml followed by a ResourceQ 1 ml column. Purification steps were performed by means of an ÄKTA Xpress system (GE Healthcare) using buffer B. The 5-LOX was eluted using buffer B supplemented with 500mM NaCl and then a gradient from 0% up to 100% of buffer B supplemented with 20 mM ATP. For the DSF experiments the buffer was changed to buffer B using an ÄKTA purifier (GE Healthcare) with three subsequentially arranged 5 ml HiTrap® Desalting Columns (GE Healtcare). The concentration was determined by performing a Bradford assay according to the Bio-Rad instruction manual.

RXRα:
For the RXRα protein expression the pMal plasmid coding for the wildtype RXRα LBD construct was transformed into competent *E.coli* T7-express cells which already held a pGro7 co-plasmid, coding for the GroEL/ES chaperone, with a chloramphenicol resistance (from the Chaperone Plasmid Set Cat. #3340, TAKARA Bio Inc. (Japan)). Positive clones were selected on LB -plates supplemented with 100 µg/ml ampicillin and 34 mg/ml chloramphenicol. These clones where then used to inoculate a pre-culture of 250 ml LB-medium supplemented with 100 µg/ml ampicillin and 34 mg/ml chloramphenicol. After incubation of 1 h (37 °C, 180 rpm), 40 ml of the pre-culture were used to inoculate the expression culture of 1 l LB-medium without antibiotics. The expression culture was incubated at 37 °C with 180 rpm. At an OD_{600} of approximately 0.6-0.7 the GroEL/ES expression was induced using 1 mg Arabinose. The flasks were then moved into a shaker at 20 °C and 120 rpm. 30 minutes later at an OD_{600} of 0.9-1.0 the target protein expression was induced using 0.5 mM IPTG. The expression cultures were incubated overnight and then harvested at 6000 rpm at 4 °C for 15 min (centrifuge: Sorval® Lynx 6000; rotor: F10-4x1000). For the purification a pellet of 2 l *E. coli* culture was suspended in 50 ml lysis buffer A supplemented with protease inhibitor, DNAse, RNAse and lysozyme. Incubation was performed for at least 30 min and the mechanically lysis was done with a cell homogenizer (Invensys APV-1000 Homogenizer, Denmark). In order to force the ATP dependent GroEL/ES release the lysate was then incubated with 1 mM ATP for at least 30 min on ice. The cell debris were removed via centrifugation (16500 x g, 20 min, 4 °C, centrifuge: Eppendorf 5810 R; rotor: F-34-6-38) and the supernatant was applied onto a 15 ml Ni-IDA Sepharose 6 fast-flow column. The target protein eluted at 300 mM imidazole. The N-terminal MBP used as a solubility-tag was removed with the help of a recombinant MBP-TEV protease during dialysis in 3 l buffer B. The TEV protease was applied with a 1:50 molar ratio. In order to remove the MBP-TEV protease, the cleaved N-Terminal MBP-tag and undigested target protein from the protein solution an amylose affinity chromatography (AAC) step was performed. An Amylose High Flow resin (NEB) on a gravity flow column was used. The MBP-TEV digest was applied onto the column and the flow through containing the cleaved protein was collected. The flow through of the AAC was then concentrated and purified in a final step with a 26/60 Superdex 75 column on an ÄKTA purifier system in assay buffer C. The elution fractions were pooled, shock frozen with liquid nitrogen and stored at -80 °C.

For the ITC measurements RXRα was dialyzed, using a dialysis membrane with a 3 kDa MWCO and placed in 2 l fresh buffer C, overnight. Then concentrated using an Amicon® Ultra-15 Centrifugal Filter Unit with a 3 kDa cutoff and centrifuging at 4°C with 1500 x g (centrifuge: Eppendorf 5810R; rotor: F-34-6-38) until a concentration higher than 50.5 µM was reached.

Table S3: All buffers used for the protein purification:

Protein	buffer A	buffer B	buffer C
sEH-H/ sEH-Fl	50 mM Tris, 500 mM NaCl, 70 mM imidazole, pH = 8	50 mM Tris, 500 mM NaCl, 400 mM imidazole, 10 mM $MgCl_2$, pH = 8	50 mM Tris, 50 mM NaCl, , pH = 8
FXR	20 mM Tris, 500 mM NaCl, 10% (V/V) glycerol, 20 mM imidazole, 0.5 mM DTT, pH = 7.5	20 mM Tris, 500 mM NaCl, 10% (V/V) glycerol, 250 mM imidazole, 0.5 mM DTT, pH = 7.5	10 mM Tris, 100 mM NaCl, 5 mM DTT, pH = 8.3
LTA4H	50 mM Tris, 500 mM NaCl, 20 mM imidazole, pH = 8	50 mM Tris, 500 mM NaCl, 400 mM imidazole, pH = 8	50 mM Tris, 50 mM NaCl, pH = 8
5-LO	50 mM Triethanolamine, 5 mM EDTA, 1 mg/ml Lysozym, 0.4 mM PMSF, 2 mM DTT, 60 µg/ml Soybean Trypsin Inhibitor, pH = 8	1X DPBS, 1 mM EDTA, pH = 7.5	1X PB, 1mM EDTA, pH = 7.5
RXRα	400 mM NaCl, 20 mM NaP_i pH 7.8, 10% (w/v) Glycerol, 20 mM ß-mercaptoethanol, 20 mM imidazole, 20 mM $MgSO_4$	400 mM NaCl, 20 mM NaP_i pH 7.8, 10% (w/v) Glycerol, 20 mM ß-mercaptoethanol	150 mM potassium fluoride, 25 mM HEPES, 5 mM DTT, 10% glycerol, pH = 7.5

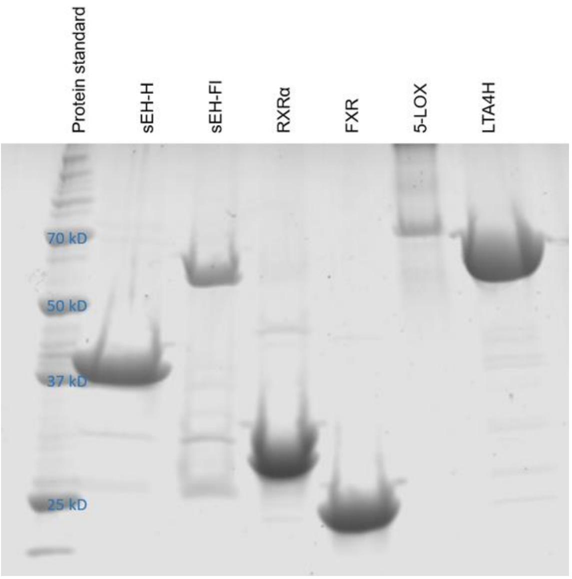

Figure S3: SDS page of the purified proteins (proteins RXRα and FXR after TEV cleavage) using a 14% polyacrylamide gel, which was stained with coomassi. Precision Plus Protein™ Unstained Protein Standards (Bio-Rad Laboratories, Inc.) was used as protein standard.

DSF screening

All experiments were performed as duplicates using MicroAmp fast 96-well plates. Each plate contained 80 different compound wells and 16 DMSO control wells. First 0.8 µl of a 25 mM Preswick Library Fragment dilution in DMSO or pure DMSO were placed in each of the well (final concentration 500 µM). Then 39.2 µl of the master mix (containing protein, Triton x-100, SYPRO® Orange Protein Gel Stain and buffer that corresponds to the tested protein) were added.

Table S4: Final concentrations in DSF experiments:

Protein	Final concentrations under screening conditions
**sEH-H**	500 µM compound, 5 µM protein, 19.625 mM Bis-tris, 3.6125 mM Tris, 3.6125 mM NaCl, 1.7% (V/V) glycerol, 0.01% (W/V) Triton X-100, 2.05 % DMSO and 2.5x SYPRO® with a pH = 7
**LTA4H**	500 µM compound, 5 µM protein, 43.5 mM Tris, 43.5 mM NaCl, 0.01% (W/V) Triton X-100, 2.05% DMSO and 2.5x SYPRO® with a pH = 8
**5-LOX**	500 µM compound, 3.5 µM protein, 0.87x DPBS, 0.87 mM EDTA, 0.01% (W/V) Triton X-100, 2.1% DMSO and 5x SYPRO® with a pH = 7.5
**RXRα**	500 µM compound, 5 µM protein, 130.5 mM potassium fluoride, 21.75 mM HEPES, 4.35 mM DTT, 8.7 % (V/V) glycerol, 0.01% (W/V) Triton X-100, 2.1% DMSO and 5x SYPRO® with a pH = 7.5
**FXR**	500 µM compound, 5 µM protein, 1.16 mM TRIS, 11.6 mM NaCl, 0.58 mM DTT, 100 mM sodium citrate , 0.01% (W/V) Triton X-100, 2.1% DMSO and 5x SYPRO® with a pH = 5.5

The measurements were performed using a iCycler iQ™ Single-Color Real Time PCR device with a heat rate of 1 °C per min, an excitation wavelength of 490 nm and an emission wavelength of 570 nm. The first derivation calculated by MyiQ Optical Software 1.0 was analyzed using Microsoft Office Excel 2013 by comparing the maxima. A shift of 1 °C (in case of 5-LOX 0.9°C) or more compared to the DMSO control were assumed as potentially binding of the fragment.

Orthogonal assay systems

Fragments:
The 10 fragments showing dual activity in the DSF experiments were validated by functional assay screening with a fixed concentration of 500 µM in case of sEH-H and LTA4H, 100 µM in case of 5-LOX and 30 µM, 10 µM and 5 µM in case of FXR and RXRα. The FXR and RXRα activity assays didn't show any results, because the concentration of used fragments was toxic for the cells or didn't show any activity. The compounds showing an inhibition greater than 50% (sEH and LTA4H), greater than 70 % (5-LOX) as well as the hits determined in the DSF experiments were further measured with concentrations between 1000 µM and 0.1 µM (sEH-H and LTA4H) and between 300 µM and 1 µM (5-LOX). The sEH and LTA4H activity assays were performed as triplicate and repeated 3 times. The 5-LOX activity assay was performed with one point per concentration and repeated 3 times Raw data was first analyzed in Microsoft Office Excel (2013), before IC_{50} curves were plotted and further analyzed using GraphPad Prism 7 and GraphPad Prism 8 for the shown graphs.

Approved drugs:
The eight approved drugs were measured in the orthogonal assay systems analogous to the fragments. The approved drugs were tested on the targets, where the fragment structure they contain, showed activity. For the sEH-H and LTA4H, they were measured as triplicate in concentrations between 1000 µM and 1 µM and each measurement was repeated three times. For the 5-LOX they were measured as single point measurement in three different experiments with concentrations between 300 µM and 1 µM. For FXR and RXRα a single hybrid reporter gene measurement with three concentrations between 30 µM and 3 µM was performed. Raw data was processed according to the fragments.

sEH-H activity assay:
The sEH-H activity assay was performed adapted from Hahn et al.[5] and Lukin et al.[6]. In short 1 µl of compound dilution in DMSO or pure DMSO (for the positive and negative control) was placed in a black 96-well plate (Thermo Scientific™ Nunc™ F96 MicroWell™ Black Polystyrene Plate) and then 89 µl of protein-mix or buffer mix (without protein as negative control) were added and the mixture was incubated for 30 min at room temperature. 10 µl of 0.6 mM 3-phenyl-cyano(6-methoxy-2-naphthalenyl)methylester-2-oxiraneacetic acid (PHOME) were added to every well. The Plate was measured every minute for 45 min. Final concentration per well was 3 nM sEH-Fl, 0.001 % Triton X-100, 25 mM Bis-tris, 0.1 mg/ml BSA, pH = 7.0, 1.6% DMSO and 60 µM PHOME.

LTA4H activity assay:
The LTA4H activity assay was performed adapted from Hiesinger et al.[3].As in the sEH-H measurement 1 µl of compound dilution in DMSO or pure DMSO (for the positive and negative control) was placed in a black 96-well plate (Thermo Scientific™ Nunc™ F96 MicroWell™ Black Polystyrene Plate) and then 89 µl of protein-mix or buffer mix (without protein as negative control) were added and incubated for 30 min at room temperature. Then the reaction was started by the addition of 10 µl 1.82 mM L-arginin-7-amido-4-methylcumarine (Arg-AMC) to every well. After the addition the plate was measured every minute for 45 min. The final concentration per well was 100 nM LTA4H, 0.001% Triton X-100, 48.7 mM Tris, 48.7 mM NaCl, pH = 8, 1.4% DMSO and 182 µM Arg-AMC.

5-LOX activity assay:
The 5-LOX-activity assay was performed adapted from Wertz et al.[9] and Kretschmer et al.[7]. In short, the purified 5-LOX protein was used in a final concentration of 3 µg/ml in assay buffer (1x PBS, 1 mM EDTA, pH = 7.4) and preincubated with 10 µl compound in DMSO or pure DMSO as positive control. Then 1 mM ATP (Roth) was added. The mixture was preincubated for 15 min on ice and then for 30 s at 37 °C using a water bath. The reaction was started by addition of 2 mM $CaCl_2$ and 20 µM arachidonic acid (Cayman). After 10 min of incubation at 37 °C the reaction was stopped by adding 1 ml of chilled methanol and the reaction vessel was placed on ice. 30 µl 1N HCl, 200 ng of Prostaglandin B1 (as internal standard) and 500 µL PBS were added. The Clean-UP C-18 solid phase extraction columns (UCT) were conditioned using 1 ml of methanol and 1 ml of water. Then the samples were given on the columns and washed with 1 ml water and 1 ml 25% methanol. The extraction was performed by the addition of 300 µl 100% methanol. The extract was diluted by adding 120 µl water and 100 µl of this dilution were analyzed by HPLC using the method and setup as described from Steinhilber et al. [10].

Hybrid reporter gene assay FXR and RXRα:
The assay was performed as described by Pollinger et al.[2]. In brief HEK293T cells were cultured at 37 °C and 5% CO_2 using a mixture of DMEM high glucose, 10% FCS, 1 mM sodium pyruvate, 100 U/ml penicillin and 100 µg/ml streptomycin. The cells were seeded in a 96-well plate with a density of $3*10^4$ cells/well and after a growth time of 24 h the medium was changed to Opti-MEM. For the transfection Lipofectamin LTX reagent (Invitrogen™) with the respective plasmids was used as described in the manufacturer's protocol. For FXR testing the plasmids pFR-Luc (Stratagene), pRL-SV40 (Promega) and pFA-CMV-hFXR-LBD were used. For RXRα the plasmids pFR-Luc (Stratagene), pRL-SV40 (Promega) and pFA-CMV-hRXRα-LBD. 5 h after transfection the medium was changed to a mixture of Opti-MEM, 100 U/ml penicillin, 100 µg/ml streptomycin and 0.1% DMSO with or without compound (untreated control). After incubation for 14-16 h the Dual-Glo™ Luciferase Assay System (Promega) was performed as described by the manufacturer. The luminescence was measured using a Spark 10M luminometer (Tecan Deutschland GmbH) device. The measured firefly luciferase data were divided by the associated renilla luciferase data and multiplied by 1000 leading to a normalized result as relative light unit (RLU). The fold activation was then obtained by dividing the RLUs of the compound by the mean of the untreated control. Every plate also contained reference compounds (GW4064 for FXR and bexarotene for RXRα) as positive control.

ITC measurements for sEH-H, LTA4H, FXR, and RXRα:
ITC experiments were performed in normal mode using an "Affinity ITC" (TA-Instruments) at 25 °C. FXR was used with a concentration of 50 µM in Tris buffer (10 mM Tris,100 mM NaCl, 5 mM DTT, pH = 8.3), supplemented with 1% DMSO and 0.5 % CHAPS. RXRα was also used in a concentration of 50 µM in a HEPES buffer (containing 150 mM potassium fluoride, 25 mM HEPES, 5 mM DTT, 10% Glycerol, pH = 7.5) supplemented with 1% DMSO and 0.5% CHAPS. LTA4H was used with a

concentration of 20 µM (for compound 10) and 40 µM (for compound 9) in a Tris buffer (50 mM Tris, 50 mM NaCl, pH = 8) supplemented with 1% of DMSO. sEH-H was used with a concentration of 20 µM (for compound 10) and 40 µM (for compound 9) in a Tris buffer (50 mM Tris, 500 mM NaCl, pH = 8) supplemented with 1% of DMSO. 300 µL of the protein solution were placed in the cell. For blank experiments the dialysis buffer supplemented with 1% DMSO and 0.5% CHAPS was used. A 250 µM or a 100 µM dilution of compound in buffer containing final concentrations of 1% DMSO (as well as 0.5 % CHAPS, in case of FXR and RXRα) was placed in the syringe. The titrations were performed with 1 injection of 0.5 µL and 30 injections with 2 µL or 35 injections with 1.5 µL as well as a spacing time between the injections of 240 or 300 seconds. One measurement with protein in the cell and blank buffer (respective buffer supplemented with 1% DMSO (as well as 0.5% CHAPS, in case of FXR and RXRα)) in the syringe was performed as well as two times for every compound measurement. A second blank experiment was performed where compound was titrated in the respective blank buffer. The results were analyzed using the program "NanoAnalyze Data Analysis" (Version 3.10.0 from TA Instruments) by subtracting a fix heat, determined by the blank experiments and using the independent option for modeling.

Results of the fragment testing

DSF experiments:

sEH-H:

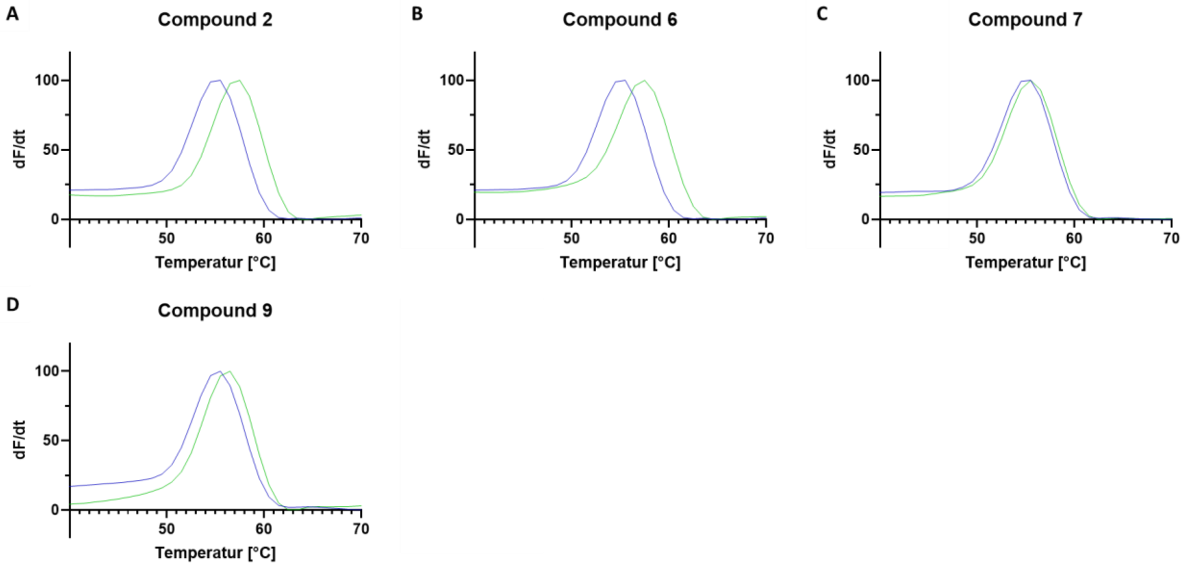

Figure S4: DSF measurement of sEH-H with 500 µM of test compounds. Exemplary shown is the first derivation of the N1 melting curves. DMSO control (blue) and compounds (green) **2**(A), **6**(B), **7**(C), and **9**(D).

LTA4H:

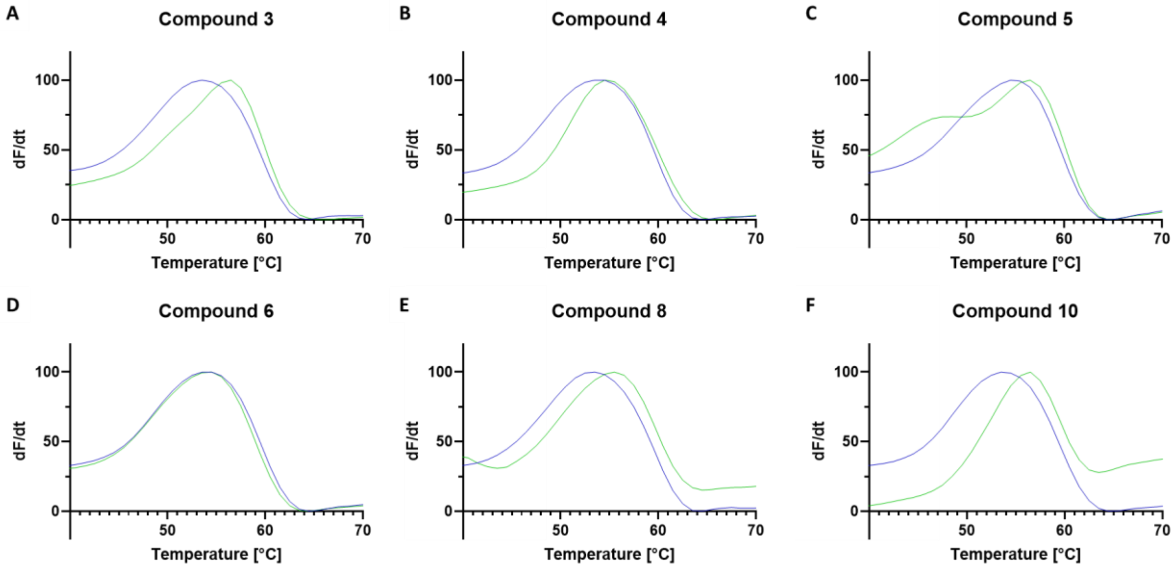

Figure S5: DSF measurement of LTA4H with 500 µM of test compounds. Exemplary shown is the first derivation of the N1 melting curves. DMSO control (blue) and compounds (green) **3**(A), **4**(B), **5**(C), **6**(D), **8**(E), and **10**(F).

5-LOX:

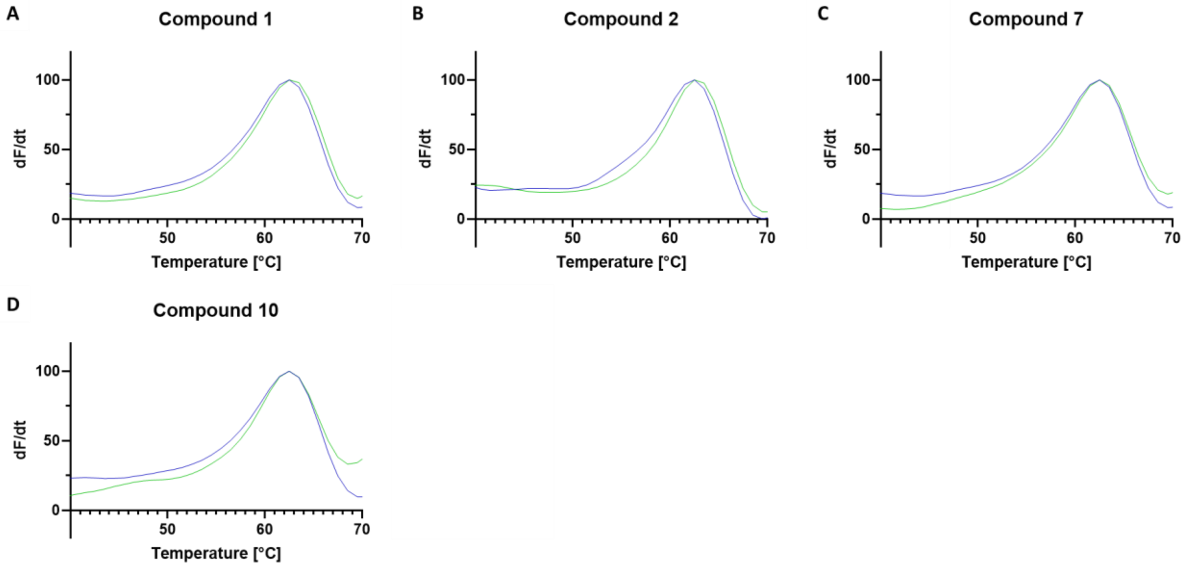

Figure S6: DSF measurement of 5-LOX with 500 µM of test compounds. Exemplary shown is the first derivation of the N1 melting curves. DMSO control (blue) and compounds (green) **1**(A), **2**(B), **7**(C), and **10** (D).

RXRα:

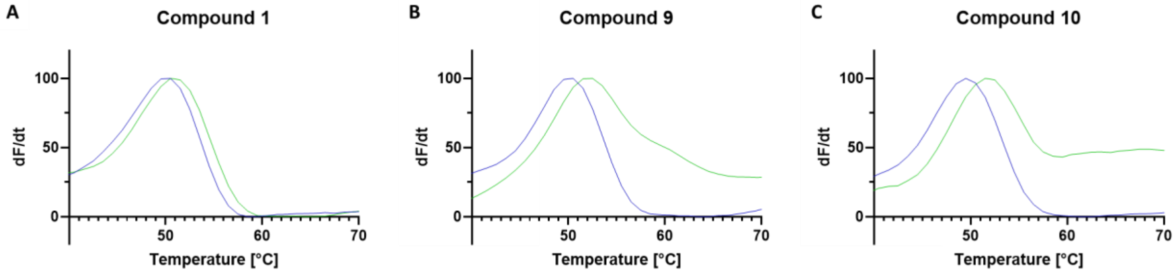

Figure S7: DSF measurement of RXRα with 500 µM of test compounds. Exemplary shown is the first derivation of the N1 melting curves. DMSO control (blue) and compounds (green) **1**(A), **9**(B) and **10**(C).

FXR:

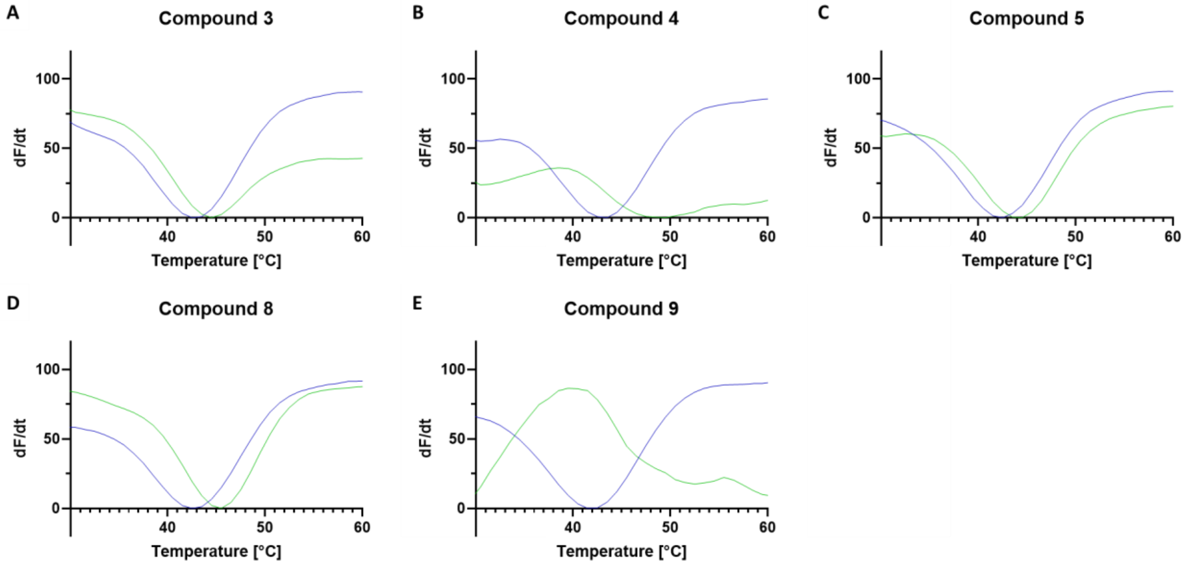

Figure S8: DSF measurement of FXR with 500 µM of test compounds. Exemplary shown is the first derivation of the N1 melting curves. DMSO control (blue) and compounds (green) **3**(A), **4**(B), **5**(C), **8**(D) and **9**(E).

IC_{50} measurement results:

sEH-H activity:

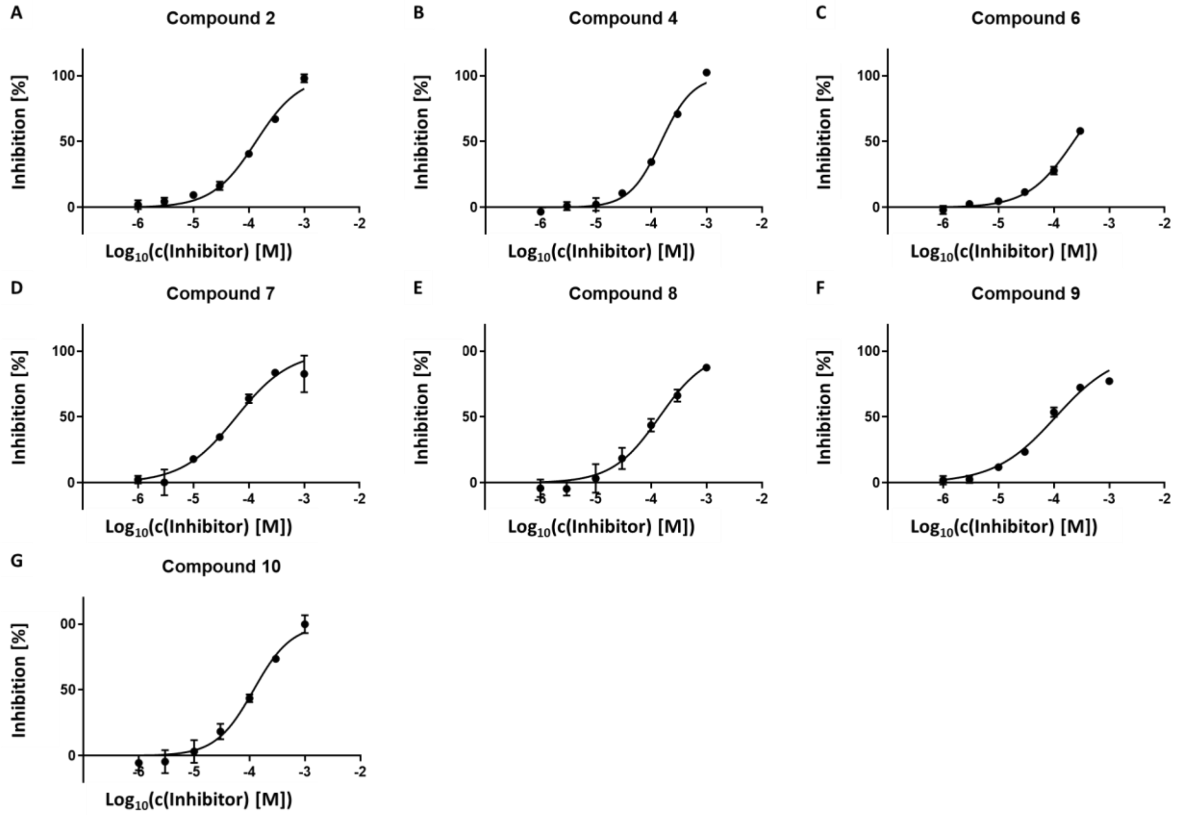

Figure S9: IC_{50} determination of compounds **2**(A), **4**(B), **6**(C), **7**(D), **8**(E), **9**(F), and **10**(G) towards sEH-H activity. Measured as triplicate in three different measurements with concentrations between 1 µM and 1000 µM.

LTA4H activity:

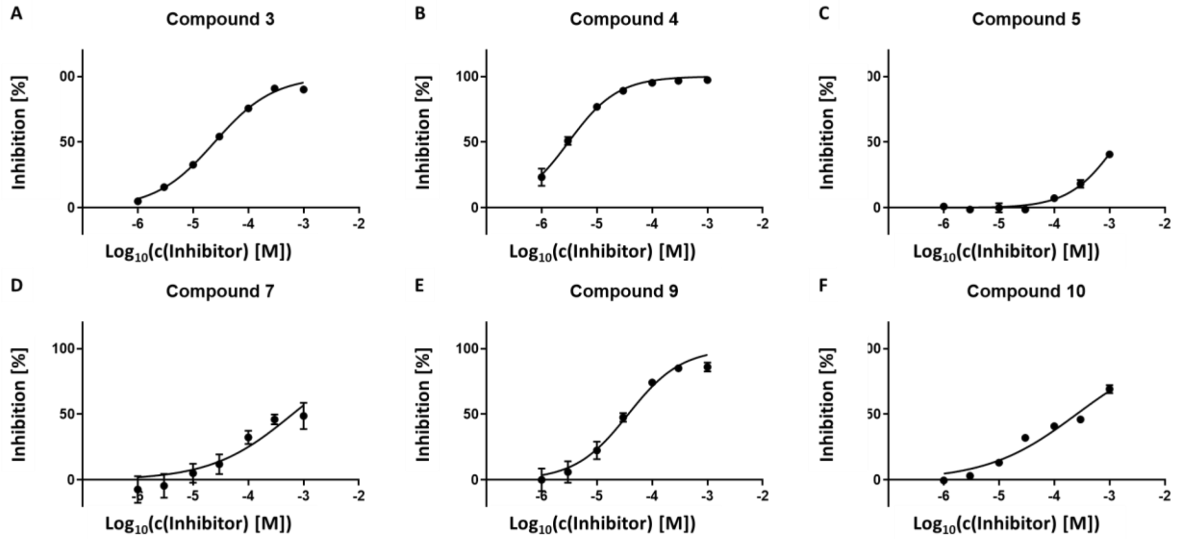

Figure S10: IC_{50} determination of compounds **3**(A), **4**(B), **5**(C), **7**(D), **9**(E), and **10**(F) towards LTA4H activity. Measured as triplicate in three different measurements with concentrations between 1 µM and 1000 µM.

5-LOX activity:

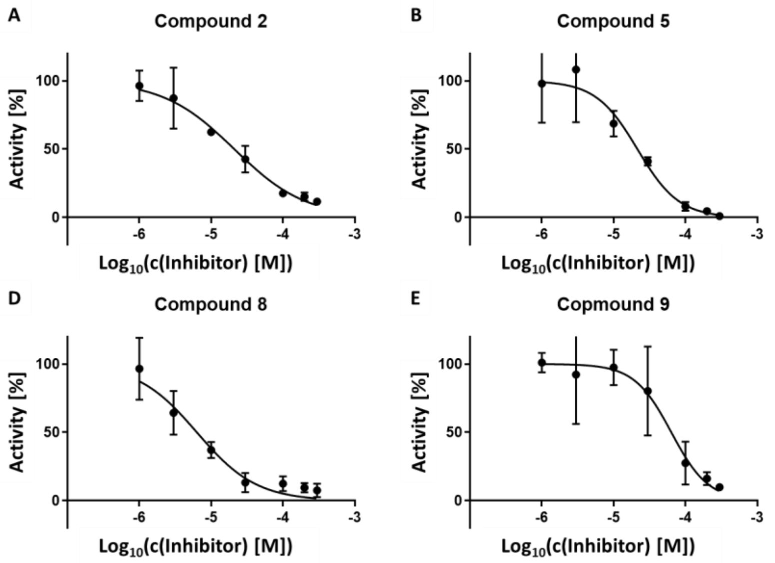

Figure S11: IC_{50} determination of compound **2**(A), **5**(B), **7**(C), **8**(D), **9**(E) and **10**(F) towards 5-LOX activity. Measured as single point in three different measurements with concentrations between 1 µM and 300 µM.

ITC Measurements results:

sEH-H measurement:

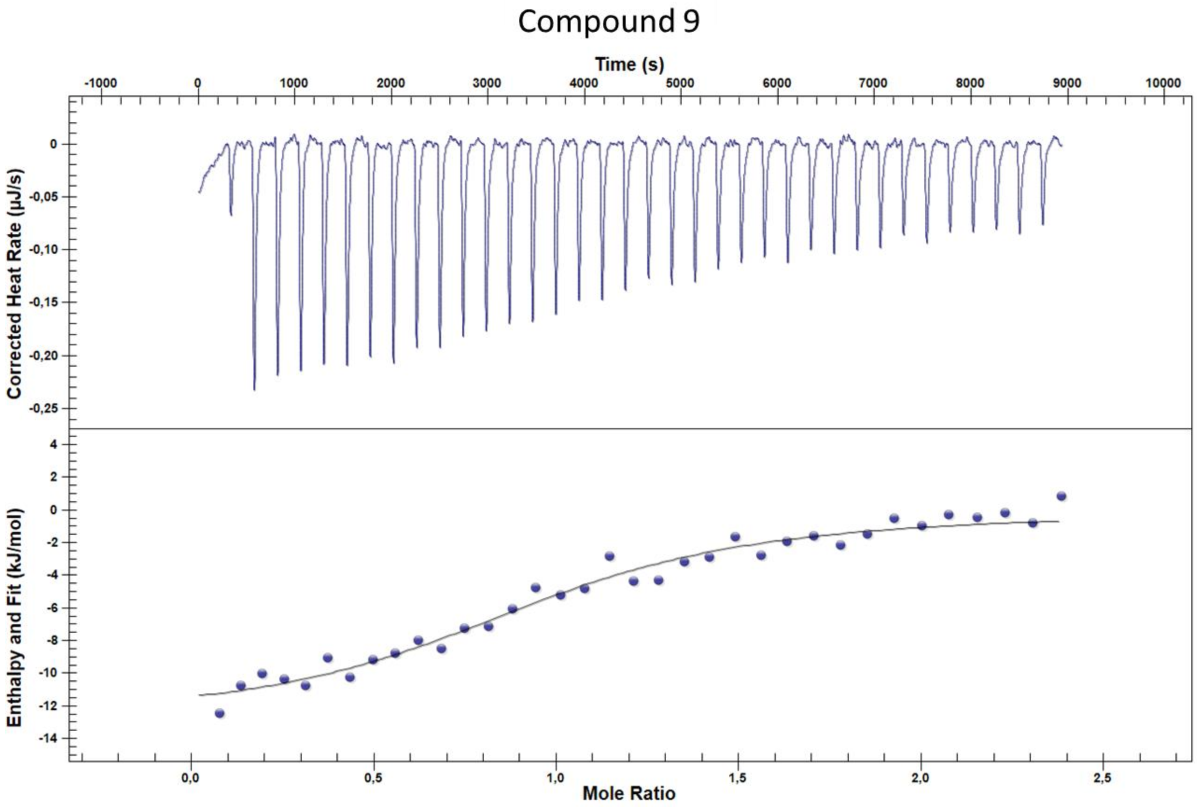

Graph S1: ITC titration of 40 µM sEH-H in cell with 250 µM compound **9** in syringe. One initial step with 0.5 µL followed by 35 steps with 1.5 µl volume. The measurement was performed at 25 °C and between every step there was a spacing of 240 s.

Compound 10

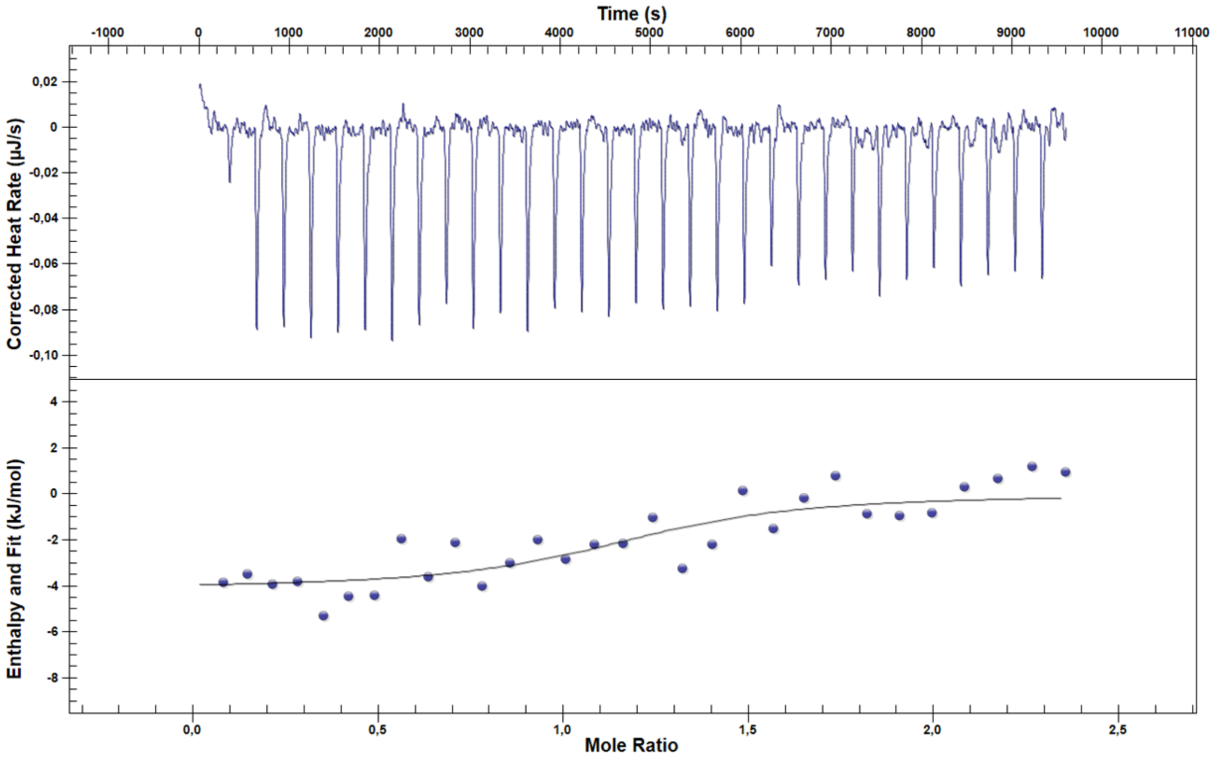

Graph S2: ITC titration of 20 µM LTA4H in cell with 100 µM compound **10** in syringe. One initial step with 0.5 µL followed by 30 steps with 2 µl volume. The measurement was performed at 25 °C and between every step there was a spacing of 300 s.

LTA4H measurement:

Compound 9

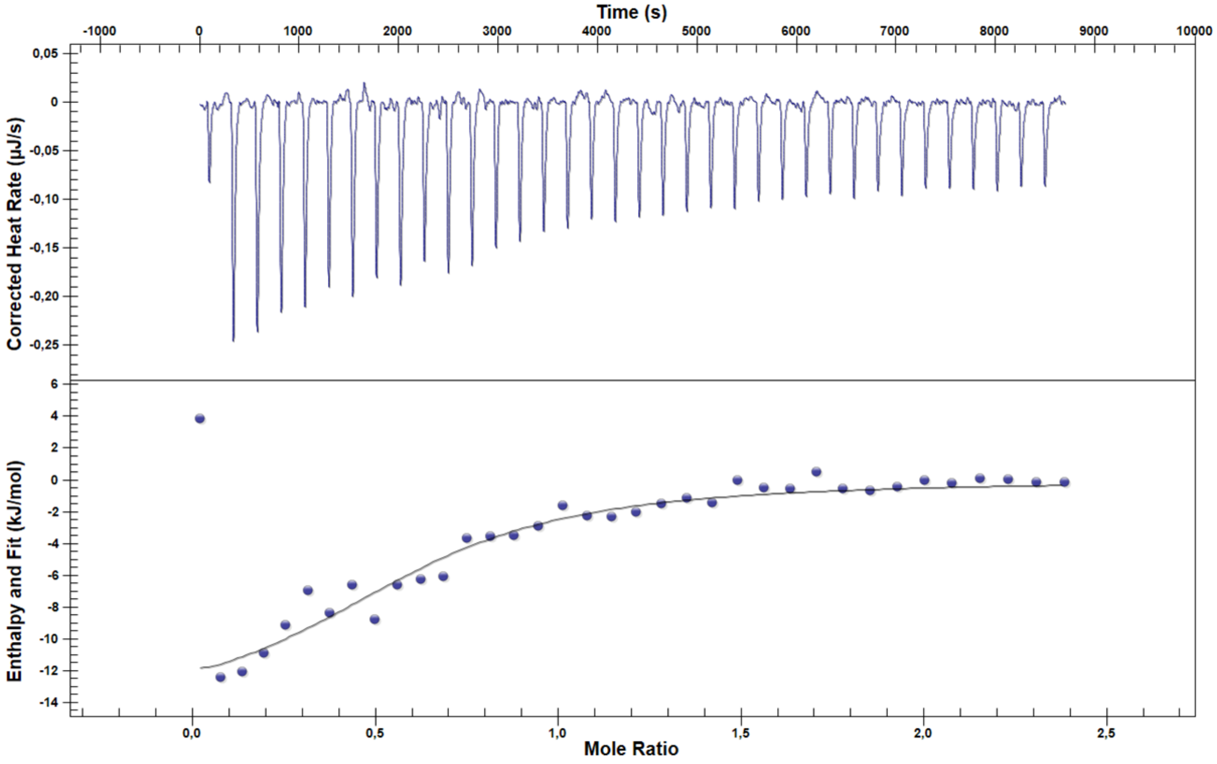

Graph S3: ITC titration of 40 µM LTA4H in cell with 250 µM compound **9** in syringe. One initial step with 0.5 µL followed by 30 steps with 1.5 µl volume. The measurement was performed at 25 °C and between every step there was a spacing of 240 s.

Compound 10

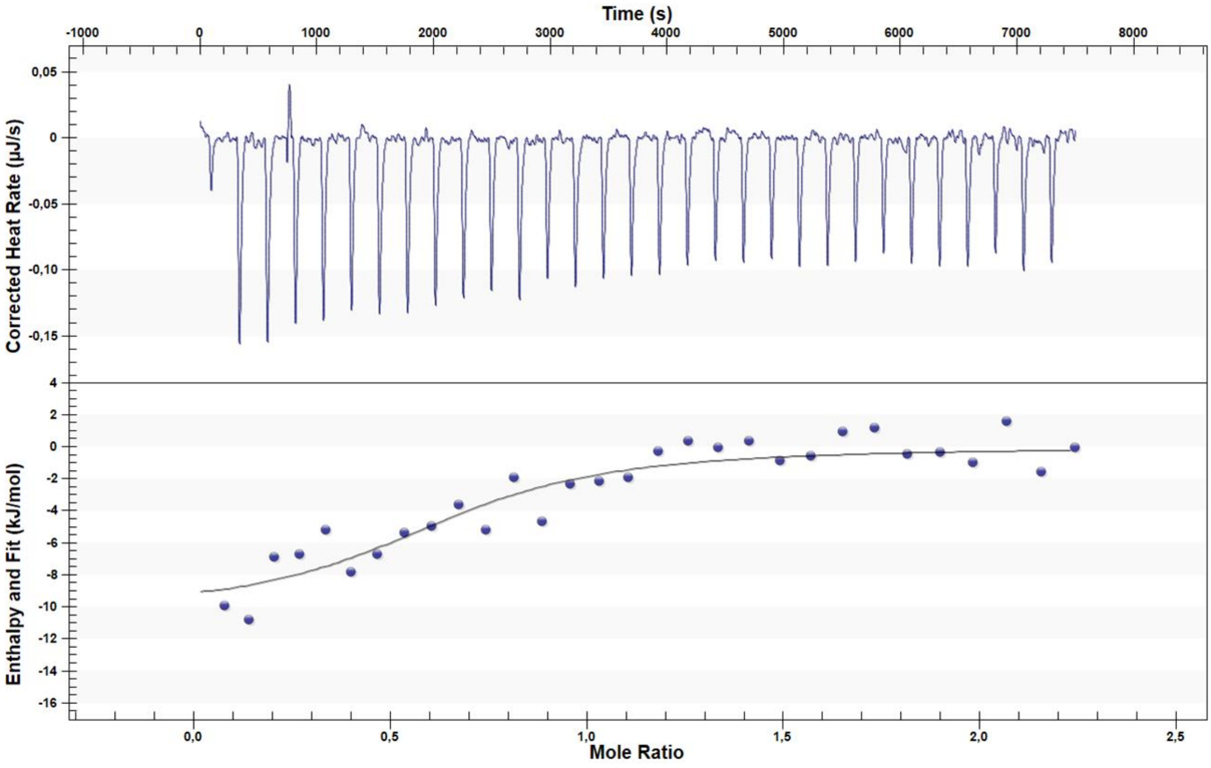

Graph S4: ITC titration of 20 µM LTA4H in cell with 100 µM compound **10** in syringe. One initial step with 0.5 µL followed by 30 steps with 2 µl volume. The measurement was performed at 25 °C and between every step there was a spacing of 240 s.

FXR measurement:

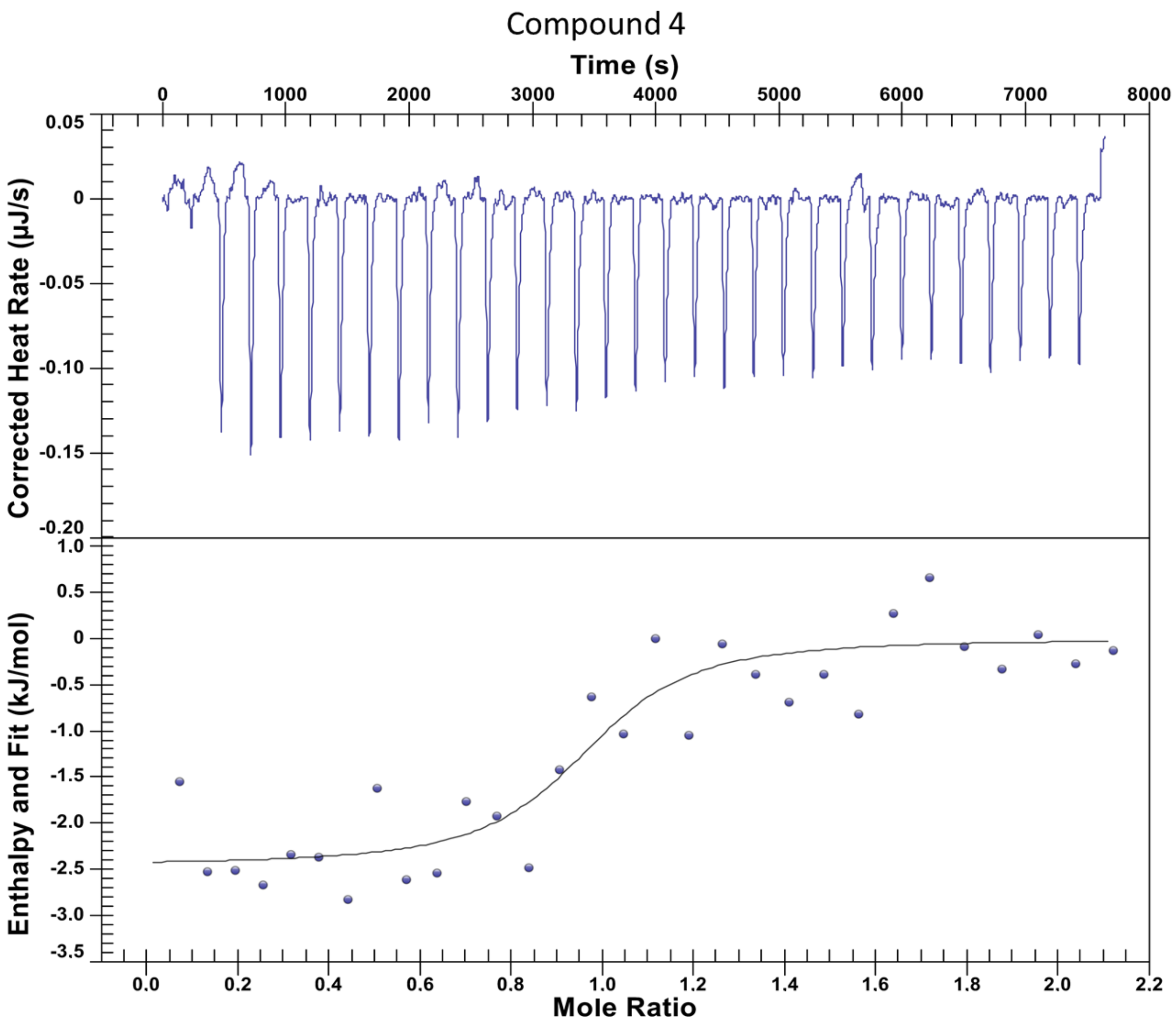

Graph S5: ITC titration of 50 µM FXR in cell with 250 µM compound **4** in syringe. One initial step with 0.5 µL followed by 30 steps with 2 µl volume. The measurement was performed at 25 °C and between every step there was a spacing of 240 s.

Compound 8

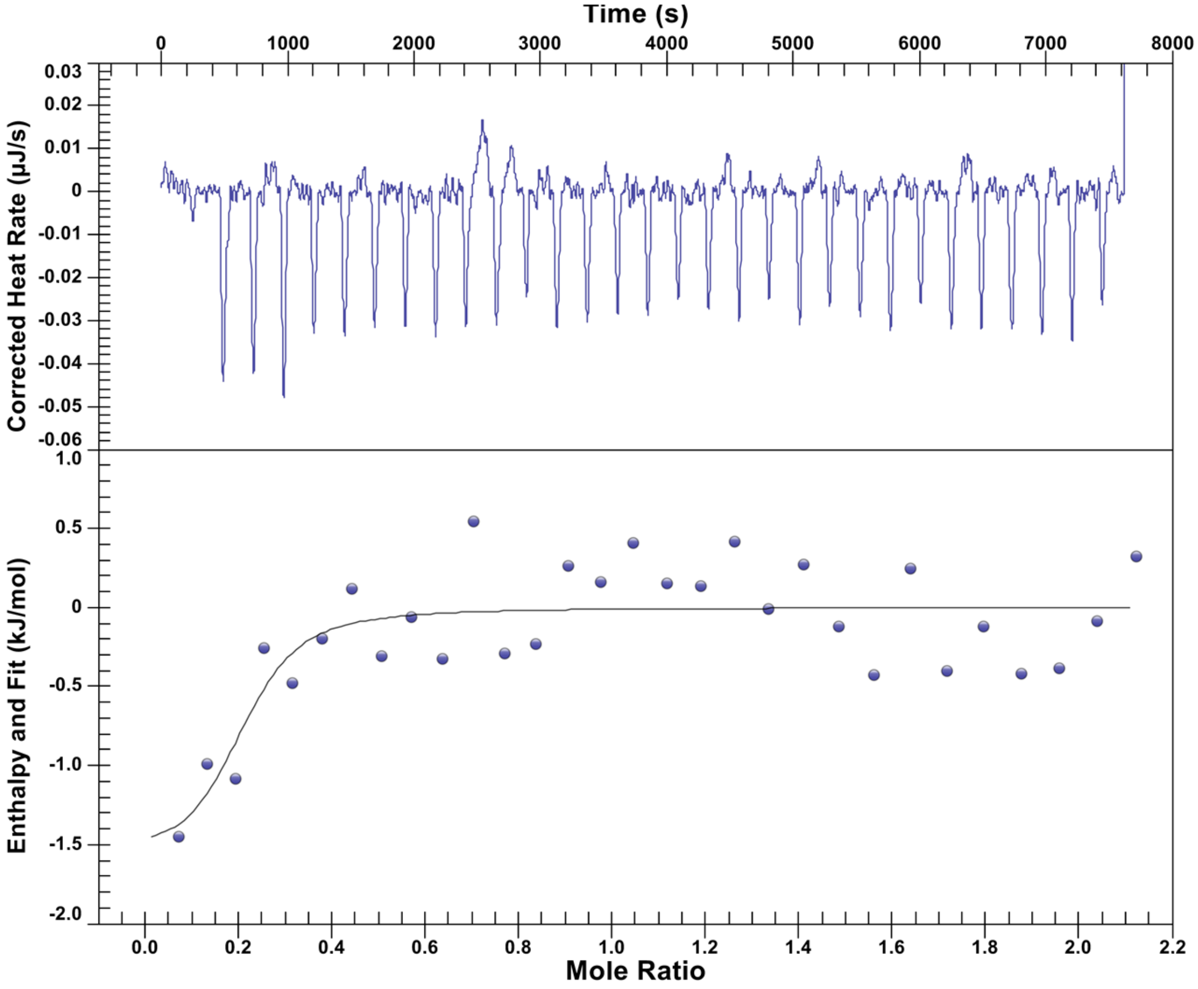

Graph S6: ITC titration of 50 µM FXR in cell with 250 µM compound **8** in syringe. One initial step with 0.5 µL followed by 30 steps with 2 µl volume. The measurement was performed at 25 °C and between every step there was a spacing of 240 s.

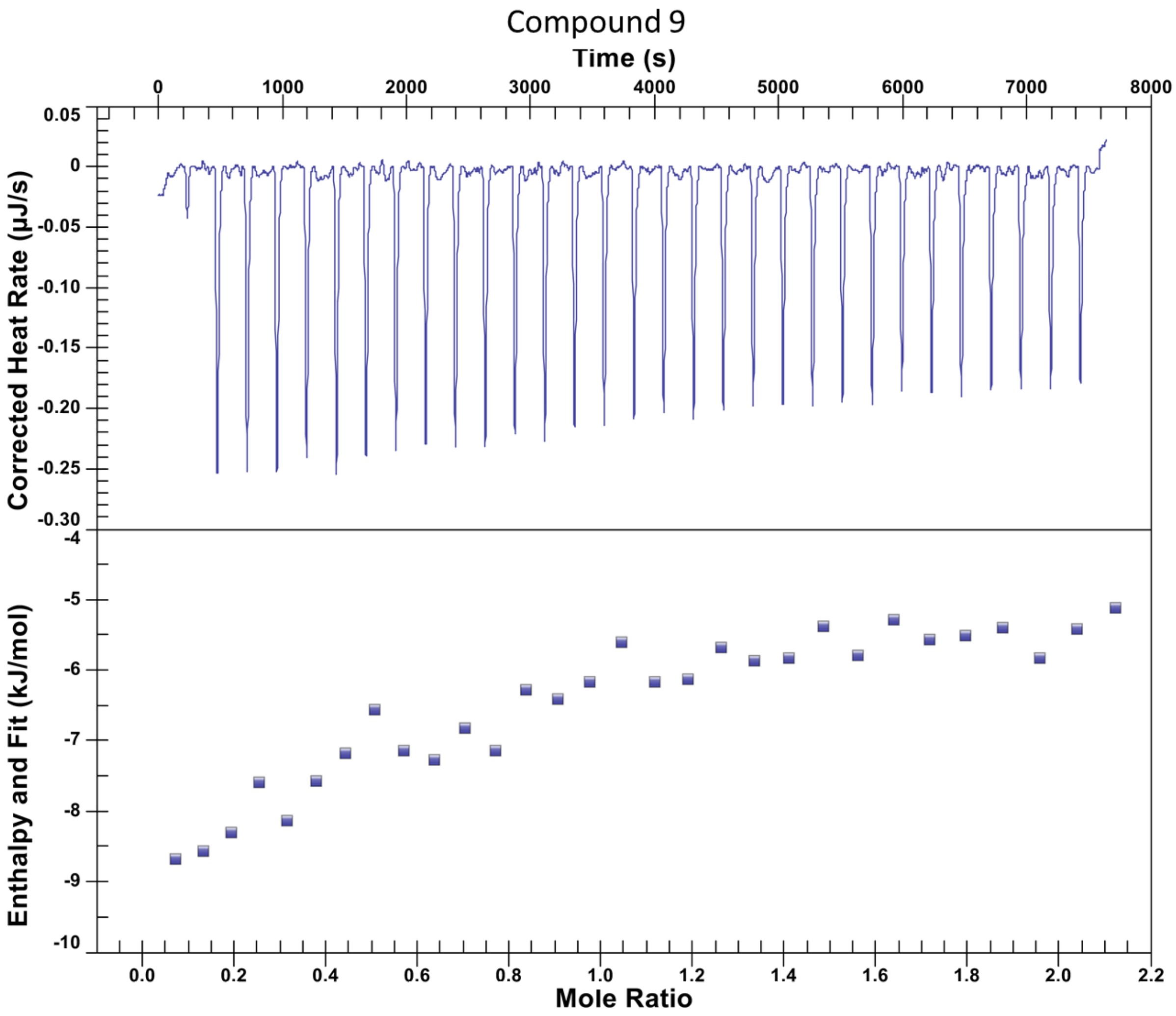

Graph S7: ITC titration of 50 µM FXR in cell with 250 µM compound **9** in syringe. One initial step with 0.5 µL followed by 30 steps with 2 µl volume. The measurement was performed at 25 °C and between every step there was a spacing of 240 s.

Results of the tested approved drugs

sEH-H activity:

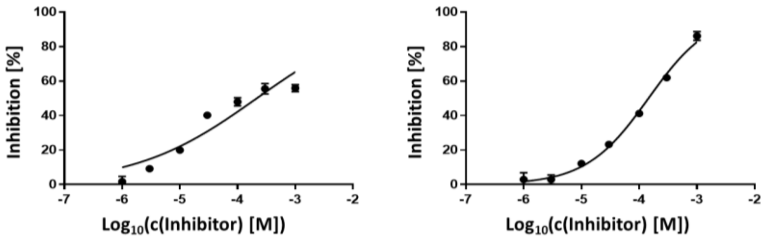

Figure S9: IC_{50} determination of compound **14** (tolvaptam, left) and **17** (diflunisal, right) towards sEH-H activity. Measured as triplicate in three different measurements with concentrations between 1 µM and 1000 µM.

LTA4H activity:

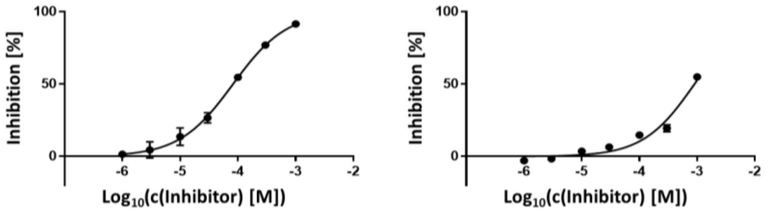

Figure S13: IC_{50} determination of compound **11** (benoxinate, left) and **18** (fluvastatin, right) towards LTA4H activity. Measured as triplicate in three different measurements with concentrations between 1 µM and 1000 µM.

5-LOX activity:

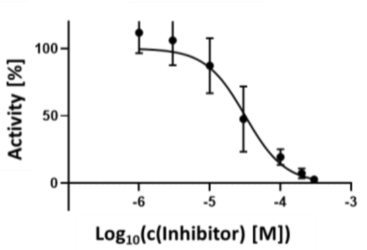

Figure S14: IC_{50} determination of compound **15** (beperidil) towards 5-LO activity. Measured as single point in three different measurements with concentrations between 1 µM and 300 µM.

Brc1ccc(cc1)C(=O)c2ccccc2
C(C1CCNCC1)c2ccccc2
C(C1NCCc2ccccc12)c3ccccc3
C(CN1CCCCC1)C(c2ccccc2)c3ccccc3
C(Cn1ccnc1)c2ccccc2
C(N1CCCC1)c2nc3ccccc3[nH]2
C(N1CCNCC1)c2ccc3OCOc3c2
C(N1CCNCC1)c2ccccc2
C(N1CCNCC1)c2cccnc2
C(Nc1ccccn1)c2ccccc2
C(Oc1ccc2OCOc2c1)[C@H]3CCCNC3
C(\C=C\c1ccccc1)N2CCNCC2
C(c1ccccc1)n2ccnc2
C1CC(CCN1)c2c[nH]cn2
C1CC(CCN1)c2ccccc2
C1CC1Nc2ncnc3[nH]cnc23
C1CCc2c(C1)[nH]c3ccccc23
C1CN(CCN1)C(c2ccccc2)c3ccccc3
C1CN(CCN1)c2ccccn2
C1CN(CCN1)c2ccncn2
C1CN(CCN1)c2ncccn2
C1CN(CCN1c2ccccc2)c3ccccc3
C1CNCCC(C1)c2ccccc2
C1CNCCN(C1)C(c2ccccc2)c3ccccc3
C1CNc2ccccc2C1
C1COC(CN1)c2ccccc2
C1COCC(N1)c2ccccc2
C1COc2ccccc2N1
C1Cc2c(CN1)[nH]c3ccccc23
C1Cc2ccccc2CCN1
C1Cc2sccc2CN1
C1Nc2ccccc2Cc3ccccc13
C1Oc2ccccc2O1
CC(=O)C1CCNCC1
CC(=O)N(CCC(=O)O)c1ccccc1
CC(=O)N1CCN(CC1)c2ccc(O)cc2
CC(=O)NCCc1c[nH]c2ccccc12
CC(=O)NCc1cccnc1
CC(=O)Nc1ccccn1
CC(=O)Nc1ncc(s1)[N+](=O)[O-]
CC(=O)Nc1nncs1
CC(=O)c1cc(Cl)ccc1O
CC(=O)c1ccc(N2CCNCC2)c(F)c1
CC(=O)c1ccc(O)cc1O
CC(=O)c1ccc2OCOc2c1
CC(=O)c1ccc2Sc3ccccc3Nc2c1
Cc1cnc(cn1)C(=O)O
Cc1n[nH]c(C)c1N
Cc1nc(cs1)c2ccccc2
Cc1nc(cs1)c2ccccc2F
Cc1nc2ccccc2[nH]1
Cc1ncc([nH]1)[N+](=O)[O-]
Cc1nccn1CCO
Cc1ncsc1CCO
Cc1nnc(N)s1
Cc1noc(N)c1C
Cc1oc(c2ccccc2)c(n1)c3ccccc3
Cc1onc(N)c1
Cc1oncc1C(=O)N
ClC1(Cl)CC1c2ccccc2
Clc1ccc(CC2=NNC(=O)c3ccccc23)cc1
Clc1ccc(CNc2ccccn2)cc1
Clc1ccc(Cn2cnc3ccccc23)cc1
Clc1ccc(OCC(=O)N2CCNCC2)cc1
Clc1ccc(cc1)C(N2CCNCC2)c3ccccc3
Clc1ccc(cc1)c2cncnc2
Clc1ccc2C(=O)c3ncccc3CCc2c1
Clc1ccc2CCc3ccccc3Nc2c1
Clc1cccc(CN2CCNCC2)c1
Clc1cccc(N2CCNCC2)c1Cl
Clc1cccc2ncccc12
Cn1c(N)nc2ccccc12
Cn1cc(CC(=O)O)c2ccccc12
Cn1nnnc1S
FC(F)(F)c1cc[nH]n1
FC(F)(F)c1ccc(cc1)C(=O)C2CCCCC2
FC(F)(F)c1nnc2CNCCn12
Fc1ccc(CN2CCNCC2)cc1
Fc1ccc(cc1)C(=O)CCCN2CCNCC2
Fc1ccc(cc1)C(N2CCNCC2)c3ccc(F)cc3
Fc1ccc(cc1)C2(CCCCC2)C#N
Fc1ccc(cc1)c2c[nH]c3ccccc23
Fc1ccc2NC(=O)Cc2c1
Fc1cccc(F)c1N2CCNCC2
Fc1ccccc1CC(=O)C2CC2
Fc1cccnc1N2CCCC2
NC(=N)CSCc1ccccc1
NC(=N)NC(=N)Nc1ccc(Cl)cc1
NC(=N)NCc1cccc(I)c1
NC(=O)C(c1ccccc1)c2ccccc2
NC(=O)CN1C(=O)c2ccccc2C1=O
NC(=O)COc1ccc(Cl)cc1

CC(=O)c1ccccc1O
CC(C(=O)O)c1cccc(F)c1
CC(C(=O)O)c1ccccc1
CC(C)(C)NCC(O)COc1ccccc1C2CCCC2
CC(C)(C)NCC(O)c1ccccc1
CC(C)(C)NCCc1ccccc1
CC(C)(C)OC(=O)\C=C\c1ccccc1
CC(C)(CN)c1ccccc1
CC(C)(Oc1ccccc1)C(=O)O
CC(C)C1NCCc2c1[nH]c3ccccc23
CC(C)CC(N)C1(CCC1)c2ccc(Cl)cc2
CC(C)NCC(O)c1ccccc1
CC(CCCN(C)C)Nc1ccncc1
CC(CO)Nc1ccccc1
CC(N)C(=O)Nc1ccccc1C
CC(N)C(=O)c1ccccc1
CC(N)COc1ccccc1
CC(O)(C(=O)O)c1ccccc1
CC(O)(c1ccccc1)c2ccccc2
CC(O)(c1ccccc1)c2ccccn2
CC(Oc1ccccc1)C(=O)N
CC1(NC(=O)NC1=O)c2ccccc2
CC1=C(O)c2ccccc2OC1=O
CC1=CC(=O)N(N1)c2ccc(C)c(C)c2
CC1=CC(=O)N(N1)c2ccccc2
CC1=CC(=O)N(N1)c2ccccc2
CC1=CC(=O)N(O)C(=C1)C2CCCCC2
CC1=CC(=O)NC(=S)N1
CC1=CC=C(C#N)C(=O)N1
CC1=CNC(=O)NC1=O
CC1CCC(=O)O1
CCC(=O)Nc1ccc(CC(=O)O)cc1
CCC(=O)Nc1ncnc2[nH]cnc12
CCC(C(=O)OCCN(CC)CC)c1ccccc1
CCC1=C(C)CN(C(=O)NCCc2ccccc2)C1=O
CCCC(=O)Nc1ccc(O)c(c1)C(=O)C
CCCC(=O)Nc1cccc(c1)C(=O)C
CCCC(=O)c1ccc(F)cc1
CCCCOc1cc(C(=O)NC)c2ccccc2n1
CCCCOc1cc(ccc1N)C(=O)OC
CCCCc1nc(Cl)c(CO)[nH]1
CCCOc1ccc(cc1N)C(=O)OCC
CCCOc1ccccc1C(=N)N
CCN(CC)C(=O)CC#N
CCN(CC)C(=O)\C=C\c1ccccc1
CCN(CC)CCNC(=O)c1ccc(N)c(Cl)c1
CCN(CC)CCNC(=O)c1ccccc1O
CCN(CC)CCNc1ccccc1
CCN(CC)CCOC(=O)C1(CCCC1)c2ccccc2
NC(=O)COc1ccccc1
NC(=O)Cc1ccccc1
NC(=O)Cc1ccccn1
NC(=O)Cc1cccs1
NC(=O)N1CCNCC1
NC(=O)NS(=O)(=O)c1ccccc1
NC(=O)c1ccc2[nH]ccc2c1
NC(=O)c1ccccc1O
NC(=O)c1ccccn1
NC(=O)c1ccncc1
NC(=O)c1nc[nH]c1N
NC1=CC=CNC1=O
NC1=Cc2ccccc2OC1=O
NC1=NC(=O)N(C=C1)[C@H]2C[C@H](O)[C@@H](CO)O2
NC1=NC(=O)N(C=N1)[C@@H]2O[C@H](CO)[C@@H](O)[C@H]2O
NC1=NCCN1
NC1CCC(=O)NC1=O
NC1CCN(Cc2ccccc2)CC1
NC1CCc2[nH]c3ccccc3c2C1
NC1CCc2ccccc12
NCC(CC(=O)O)c1ccccc1
NCC(O)COc1cccc2ccccc12
NCC1(CC1)c2ccccc2
NCCCCN1CCN(CC1)c2ncccn2
NCCCCc1ccccc1
NCCCOc1cccc(CN2CCCCC2)c1
NCCOC(c1ccc(F)cc1)c2ccc(F)cc2
NCCOCc1ccccc1
NCCc1c[nH]c2ccc(O)cc12
NCCc1c[nH]cn1
NCCc1ccc(F)cc1F
NCCc1ccc(O)cc1
NCCc1ccc(cc1)S(=O)(=O)N
NCc1c(N)ccc(Cl)c1Cl
NCc1cscn1
NCc1ncc[nH]1
NNC(=O)c1ccccc1
NNC(=O)c1ccncc1
NNCCc1ccccc1
NS(=O)(=O)c1cc(ccc1Cl)C(=O)O
NS(=O)(=O)c1ccc(Cl)cc1
NS(=O)(=O)c1ccc(cc1)C(=O)O
NS(=O)(=O)c1ccccc1
NS(=O)(=O)c1ccccc1C(F)(F)F
N[C@@H](C(=O)N)c1ccccc1
Nc1c(Cl)ccc2nsnc12
Nc1ccc(CS(=O)(=O)N2CCCC2)cc1
Nc1ccc(Cl)nn1
Nc1ccc(N)c(N)n1

CCN(CC)CCOC(=O)c1ccccc1
CCN(CC)CCOc1ccc(cc1)C(=O)C
CCN(CC)CCOc1ccccc1O
CCN(CC)Cc1cc(N)ccc1O
CCN(CC)S(=O)(=O)c1ccccc1
CCN(CCCCO)C(C)Cc1ccc(OC)cc1
CCN(Cc1ccccc1)c2ccccc2
CCN1CCCC1CN
CCN1CCCC1CNC(=O)c2ccc(N)cc2
CCNC(=O)c1ccc(Cl)cc1
CCNC(=O)c1cccnc1
CCOC(=O)C1c2ccccc2Oc3ccccc13
CCOC(=O)c1cccnc1
CCOc1ccc(N)cc1
CCOc1ccccc1C(=N)N
CCOc1ccccc1O
CCOc1ccccc1OCC2CNCCO2
CCOc1ccccn1
CCc1ccc(CCO)nc1
CCc1cccc2c(CCO)c[nH]c12
CN(C)C(=O)C(c1ccccc1)c2ccccc2
CN(C)C(=O)Oc1ccccc1
CN(C)C(c1ccccc1)c2ccccc2
CN(C)CCCC(=O)c1ccc(F)cc1
CN(C)CCNC(=O)c1ccc(N)cc1
CN(C)CCNCc1ccccc1
CN(C)Cc1oc(CSCCN)cc1
CN1C(=O)CCC1=O
CN1C(=O)Nc2nc[nH]c2C1=O
CN1C2CCC1CC(O)C2
CN1C=CC=CC1=O
CN1CC(c2ccccc2)c3cccc(N)c3C1
CN1CCC[C@@H]1c2cccnc2
CN1CCN(CC1)C(c2ccccc2)c3ccccc3
CN1CCN(CC1)S(=O)(=O)c2ccccc2
CN1CCN(CC1)c2cc(N)ncn2
CN1CCN(CC1)c2ccc(N)cc2
CN1CCN(CC1)c2ccccc2
CN1CCN(CC1)c2nccc(N)n2
CN1CCN(CCCC(=O)c2ccc(F)cc2)CC1
CN1CCNCC1=O
CN1CCc2cccc(N)c2C1
CN1N(C(=O)C(=C1C)N)c2ccccc2
CN1NC(=O)C=C1
CNC(=O)c1c(C)onc1c2ccccc2
CNC(=O)c1c(OC)ccc(Br)c1OC
CNC(=O)c1cc(Cl)c(N)cc1OC
CNC(=O)c1ccc(Cl)cc1
CNC(=O)c1cnc(C)cn1
Nc1ccc(cc1Cl)[N+](=O)[O-]
Nc1ccc2CCCCc2c1
Nc1cccc(Nc2ncccn2)c1
Nc1cccc(O)c1
Nc1cccc(c1)C#C
Nc1cccc(c1)C(F)(F)F
Nc1ccccc1S(=O)(=O)N
Nc1ccccn1
Nc1cccnn1
Nc1ccnc2ccccc12
Nc1ccnn1c2ccccc2
Nc1cnc2ccccc2n1
Nc1nc(N)nc(n1)c2ccccc2
Nc1nc2CCCCc2s1
Nc1nc2cc(ccc2[nH]1)C(=O)c3ccccc3
Nc1nc2ccccc2n1Cc3ccc(F)cc3
Nc1ncc(s1)[N+](=O)[O-]
Nc1ncnc2c1ncn2CCO
Nc1ncns1
Nc1nncs1
Nc1oc2ccccc2n1
O=C(C1COc2ccccc2O1)N3CCNCC3
O=C(CC#N)N1CCCCC1
O=C(CCN1CCCCC1)c2ccccc2
O=C(Cc1ccccc1)c2ccccc2
O=C(N1CCNCC1)c2ccccc2
O=C(c1ccccc1)c2ccccc2
O=C1CC2(CCCC2)CC(=O)N1
O=C1CCCc2[nH]ccc12
O=C1CCN(CCc2ccccc2)CC1
O=C1CCNc2ccccc2N1
O=C1CCOc2ccccc12
O=C1CCc2ccccc2N1
O=C1CN(Cc2ccccc2)CCN1
O=C1CNCCN1
O=C1CNCN1
O=C1COCCN1
O=C1COc2ccccc12
O=C1N(Cc2ccccc12)c3ccccc3
O=C1NC=CC=C1C#N
O=C1NC=NC=C1c2ccccc2
O=C1NCCc2ccccc12
O=C1NCN(c2ccccc2)C13CCNCC3
O=C1NN=C(Cc2ccccc2)c3ccccc13
O=C1NN=C2C=CC=CN12
O=C1NN=Cc2ccccc12
O=C1Nc2ccccc2N1C3CCNCC3
O=C1Nc2ccccc2Nc3ccccc13
O=C1Nc2ccccc2O1

CNC(C)(C)Cc1ccccc1
CNCC(c1ccc(OC)cc1)C2(O)CCCCC2
CNCCC(c1ccccc1)c2ccccc2
CNCCN(C)C(c1ccccc1)c2ccccc2
CNCCc1c[nH]c2ccccc12
CNCCc1ccc(OC)c(OC)c1
CNS(=O)(=O)CCc1ccccc1
CNS(=O)(=O)Cc1ccc2[nH]cc(CCN)c2c1
CNS(=O)(=O)c1ccccc1
COC(=O)C(CO)c1ccccc1
COC(=O)C(O)(C1CCCC1)c2ccccc2
COC(=O)C(O)(C1CCCCC1)c2ccccc2
COC(=O)C(O)c1ccccc1
COC(=O)C(c1ccccc1)c2ccccc2
COC(=O)C1=C(C)NC(=C(C1)C(=O)OC)C
COC(=O)CN1CCc2sccc2C1
COC(=O)Nc1ccccc1
COC(=O)c1c[nH]c2ccccc12
COC(=O)c1ccc(N)cc1
COC(=O)c1scc(C)c1N
COCCOC(=O)c1ccc(N)cc1
COc1cc(C)cc(C)c1C
COc1cc(N)c(Cl)cc1C(=O)NC2CCNCC2
COc1cc(N)ncn1
COc1cc(NS(=O)(=O)c2ccc(N)cc2)ncn1
COc1cc(cc(OC)c1OC)C(=O)N
COc1cc2CCN(C)C(C)c2cc1OC
COc1cc2CCN(C)Cc2cc1OC
COc1cc2CCNC(=O)Cc2cc1OC
COc1cc2CCNC(C)c2cc1OC
COc1cc2CCNCc2cc1OC
COc1cc2[C@H](C)NCCc2cc1O
COc1cc2ccncc2cc1OC
COc1ccc(C(=O)C)c(O)c1
COc1ccc(CC(=O)O)cc1
COc1ccc(CC(C)N)cc1
COc1ccc(CCN(C)C)cc1OC
COc1ccc(CCN)cc1O
COc1ccc(CCN)cc1OC
COc1ccc(CCN2CCC(N)CC2)cc1
COc1ccc(CN2CCNCC2)cc1
COc1ccc(CNCCN(C)C)cc1
COc1ccc(CNc2ncccn2)cc1
COc1ccc(Cc2ccc(OC)cc2)cc1
COc1ccc(Cl)cc1C(=O)N
COc1ccc(N)nn1
COc1ccc(O)cc1O
COc1ccc(OC)c(c1)C(C)O
COc1ccc(OCC(=O)N)cc1
O=C1OCc2ccccc12
O=C1Oc2ccccc2C=C1
OC(=O)C(c1ccccc1)c2ccccc2
OC(=O)C1(CCCC1)c2ccccc2
OC(=O)C1=CC(=O)c2ccccc2O1
OC(=O)CC(c1ccccc1)c2ccccc2
OC(=O)CC1CCCCC1
OC(=O)CCC(=O)Nc1ccccc1
OC(=O)CCC(=O)c1ccccc1
OC(=O)CCCc1ccc(cc1)c2ccccc2
OC(=O)CC[C@H](NC(=O)c1ccccc1)C(=O)O
OC(=O)CCc1ccc(Cl)cc1
OC(=O)CCc1oc(cn1)c2ccccc2
OC(=O)CNC(=O)c1ccccc1
OC(=O)COc1ccccc1
OC(=O)Cc1ccccc1
OC(=O)Cc1cccs1
OC(=O)\C=C\c1cnc[nH]1
OC(=O)c1cc(ccc1O)c2ccc(F)cc2
OC(=O)c1cc(ccc1O)c2ccccc2
OC(=O)c1ccc(Oc2ccccc2)cc1
OC(=O)c1ccc(cc1)C(F)(F)F
OC(=O)c1cccc(c1)c2ccc(F)cc2F
OC(=O)c1ccccc1NCc2occc2
OC(=O)c1ccccc1Nc2cccc(Cl)c2
OC(=O)c1ccccc1O
OC(=O)c1ccccn1
OC(=O)c1cccnc1Nc2cccc(Cl)c2
OC(=O)c1cccnc1Nc2ccccc2
OC(=O)c1cnccn1
OC(C1CCNCC1)(c2ccccc2)c3ccccc3
OC(Cn1ccnc1)c2ccc(Cl)cc2Cl
OC(c1ccc(Cl)cc1)c2ccccn2
OC(c1ccc(F)cc1)c2ccc(F)cc2
OC(c1ccccc1)c2ccc(Cl)cc2
OC(c1ccccc1)c2ccccc2
OC(c1ccccc1)c2ccccn2
OC1(CCNCC1)c2ccc(Cl)cc2
OC1=CC(=O)Oc2ccccc12
OC1=CNS(=O)(=O)c2ccsc12
OC1CCN(CC1)c2ccccc2F
OC1N(C(=O)c2nccnc12)c3ccc(Cl)cn3
OC1c2ccccc2CCc3ccccc13
OCC(C(=O)O)c1ccccc1
OCC(O)COc1ccc(Cl)cc1
OCC(O)COc1ccccc1
OCC1CCCNC1
OCC1CCNCC1
OCCCN1CCN(CC1)c2ccccc2

COc1ccc(cc1)C(F)(F)F
COc1ccc(cc1)N2CCN(CC2)c3ccc(N)cc3
COc1ccc(cc1)S(=O)(=O)N2CCNCC2
COc1ccc(cc1N2CCCC2)C(=O)O
COc1ccc2C(=O)COc2c1
COc1ccc2CC(CCc2c1)C(=O)O
COc1ccc2CCCCc2c1
COc1ccc2[nH]c(SC)nc2c1
COc1ccc2[nH]cc(CCN)c2c1
COc1ccc2[nH]cnc2c1
COc1ccc2cc[nH]c2c1
COc1ccc2ccncc2c1
COc1ccc2nc(N)sc2c1
COc1cccc2C(=O)CCCc12
COc1ccccc1N2CCN(CCCN)CC2
COc1ccccc1OCCCO
COc1ccccc1OCCO
CS(=O)(=O)NC(Cc1ccccc1)C(=O)O
CS(=O)(=O)Nc1ccccc1
CS(=O)(=O)Nc1ccccc1Oc2ccccc2
CS(=O)(=O)c1ccc(CO)cc1
CS(=O)(=O)c1ccc(N)cc1
C[C@@H](O)c1cc(cc(c1)C(F)(F)F)C(F)(F)F
C[C@H]1NC(=O)NC1=O
C[N+](C)(CCO)Cc1ccccc1
Cc1[nH]c2ccccc2c1CC(=O)O
Cc1[nH]cnc1CO
Cc1c(C)c2OC(C)(C)CCc2c(C)c1O
Cc1c(N)cccc1C(F)(F)F
Cc1c[nH]nc1C
Cc1cc(C)nc(O)n1
Cc1cc(cc(C)c1CC2=NCCN2)C(C)(C)C
Cc1cc(ccc1O)C(C)(C)CC(C)(C)C
Cc1cc(nnc1N)c2ccccc2
Cc1ccc(N)c(c1)C#N
Cc1ccc(cc1)S(=O)(=O)N
Cc1ccc(cc1)S(=O)(=O)NC(=O)N
Cc1cccc(C)c1N
Cc1cccc(C)c1NC(=O)CN2CCNCC2
Cc1cccc(Nc2ccccc2C(=O)O)c1
Cc1cccc(O)c1
Cc1ccccc1C(=O)Nc2ccccc2
Cc1ccccc1C(O)c2ccccc2
Cc1ccccc1Nc2ncccc2C(=O)O
Cc1cn(cn1)c2cc(N)cc(c2)C(F)(F)F
OCCN1CCN(CC1)c2ccccc2
OCCOc1cccc2[nH]ccc12
OCCc1ccc(Cl)cc1
OCCn1ccnc1
OCCn1cncn1
OC[C@H]1O[C@@H](C[C@@H]1O)N2C=CC(=O)NC2=O
OCc1cc(O)cc(O)c1
OCc1cc2ccccc2[nH]1
OCc1ccc(F)cc1F
OCc1ccc(O)cc1
OCc1ccc(cc1)[N+](=O)[O-]
OCc1ccc2OCOc2c1
OCc1cccnc1
OCc1ccncc1
ONC(=O)Cc1ccccc1
Oc1cc(Cl)c(Cl)cc1Cl
Oc1ccc(cc1)C(=O)NCc2cccnc2
Oc1ccc(cc1)c2ccc(F)cc2F
Oc1ccc2C(=O)CC(Oc2c1)c3ccccc3
Oc1ccc2C(=O)COc2c1
Oc1ccc2CCC(=O)Nc2c1
Oc1ccc2CCCc2c1
Oc1ccc2[nH]ccc2c1
Oc1ccc2cc[nH]c2c1
Oc1cccc(CN2CCCCC2)c1
Oc1cccc2CCCCc12
Oc1cccc2[nH]ccc12
Oc1cccc2cccnc12
Oc1ccccc1C(=O)CCc2ccccc2
Oc1ccccc1C(=O)Nc2ccccc2Cl
Oc1ccccc1C(=O)OC2CCCCC2
Oc1ccccc1C2CCCC2
Oc1ccccc1N2CCNCC2
Oc1ccccc1OCC(F)(F)F
Oc1nsnc1N2CCOCC2
Sc1nc2ccccc2[nH]1
Sc1ncc[nH]1
c1ccc(cc1)C(c2ccccc2)n3ccnc3
c1ccc(cc1)C(c2ccccc2)n3cncn3
c1ccc(cc1)c2c[nH]cn2
c1ccc(cc1)c2ccccc2c3nnn[nH]3
c1ccc(cc1)c2nnn[nH]2
c1ccc(cc1)n2ccc3ccccc23
c1ccc(cc1)n2ccnc2
c1ccc2cnccc2c1
c1ccc2nccnc2c1
c1cnc2[nH]ncc2c1
c1cnccn1

References

(1) Baell, J. B.; Holloway, G. A. New substructure filters for removal of pan assay interference compounds (PAINS) from screening libraries and for their exclusion in bioassays. *Journal of medicinal chemistry* **2010**, *53*, 2719–2740.

(2) Pollinger, J.; Schierle, S.; Gellrich, L.; Ohrndorf, J.; Kaiser, A.; Heitel, P.; Chaikuad, A.; Knapp, S.; Merk, D. A Novel Biphenyl-based Chemotype of Retinoid X Receptor Ligands Enables Subtype and Heterodimer Preferences. *ACS medicinal chemistry letters* **2019**, *10*, 1346–1352.

(3) Hiesinger, K.; Schott, A.; Kramer, J. S.; Blöcher, R.; Witt, F.; Wittmann, S. K.; Steinhilber, D.; Pogoryelov, D.; Gerstmeier, J.; Werz, O.*; et al.* Design of Dual Inhibitors of Soluble Epoxide Hydrolase and LTA4 Hydrolase. *ACS medicinal chemistry letters* **2020**, *11*, 298–302.

(4) Merk, D.; Sreeramulu, S.; Kudlinzki, D.; Saxena, K.; Linhard, V.; Gande, S. L.; Hiller, F.; Lamers, C.; Nilsson, E.; Aagaard, A.*; et al.* Molecular tuning of farnesoid X receptor partial agonism. *Nature communications* **2019**, *10*, 2915.

(5) Hahn, S.; Achenbach, J.; Buscató, E.; Klingler, F.-M.; Schroeder, M.; Meirer, K.; Hieke, M.; Heering, J.; Barbosa-Sicard, E.; Loehr, F.*; et al.* Complementary screening techniques yielded fragments that inhibit the phosphatase activity of soluble epoxide hydrolase. *ChemMedChem* **2011**, *6*, 2146–2149.

(6) Lukin, A.; Kramer, J.; Hartmann, M.; Weizel, L.; Hernandez-Olmos, V.; Falahati, K.; Burghardt, I.; Kalinchenkova, N.; Bagnyukova, D.; Zhurilo, N.*; et al.* Discovery of polar spirocyclic orally bioavailable urea inhibitors of soluble epoxide hydrolase. *Bioorganic chemistry* **2018**, *80*, 655–667.

(7) M Kretschmer, S. B.; Woltersdorf, S.; Rödl, C. B.; Vogt, D.; Häfner, A.-K.; Steinhilber, D.; Stark, H.; Hofmann, B. Development of novel aminothiazole-comprising 5-LO inhibitors. *Future medicinal chemistry* **2016**, *8*, 149–164.

(8) Schröder, M.; Häfner, A.-K.; Hofmann, B.; Rådmark, O.; Tumulka, F.; Abele, R.; Dötsch, V.; Steinhilber, D. Stabilisation and characterisation of the isolated regulatory domain of human 5-lipoxygenase. *Biochimica et biophysica acta* **2014**, *1842*, 1538–1547.

(9) Werz, O.; Szellas, D.; Henseler, M.; Steinhilber, D. Nonredox 5-lipoxygenase inhibitors require glutathione peroxidase for efficient inhibition of 5-lipoxygenase activity. *Molecular pharmacology* **1998**, *54*, 445–451.

(10) Steinhilber, D.; Herrmann, T.; Roth, H. J. Seperation of lipoxines and leukotrienes from granuocytes. *Journal of Chromattography* **1989**, *493*, 361–366.
